# Intermittent fasting alters the antigen-specific CD5+ B-1 cell natural antibody repertoire in male and female mice

**DOI:** 10.1093/immhor/vlag038

**Published:** 2026-07-17

**Authors:** Sarah E Webster, Jordan Terry, Naomi L Tsuji, Daken Heck, Nichol E Holodick

**Affiliations:** Center for Immunobiology and the Department of Investigative Medicine, Western Michigan University Homer Stryker M.D. School of Medicine, Kalamazoo, MI, United States; Center for Immunobiology and the Department of Investigative Medicine, Western Michigan University Homer Stryker M.D. School of Medicine, Kalamazoo, MI, United States; Center for Immunobiology and the Department of Investigative Medicine, Western Michigan University Homer Stryker M.D. School of Medicine, Kalamazoo, MI, United States; Center for Immunobiology and the Department of Investigative Medicine, Western Michigan University Homer Stryker M.D. School of Medicine, Kalamazoo, MI, United States; Center for Immunobiology and the Department of Investigative Medicine, Western Michigan University Homer Stryker M.D. School of Medicine, Kalamazoo, MI, United States

**Keywords:** B cells, B-1 cells, intermittent fasting, mouse, natural antibody

## Abstract

Natural antibodies (NAbs), primarily produced by CD5+ B-1 cells, provide critical early protection against infections such as *Streptococcus pneumoniae*. The structure of these natural antibodies, germline-like with minimal N-additions, is essential for their protective capacity. The protective capacity and germline status of NAbs are compromised in aged male mice but maintained in aged female mice. CD5+ B-1 cells are maintained through self-renewal, necessary for maintaining germline-like natural antibodies, and this process depends on autophagy. Intermittent fasting enhances autophagy and stem cell self-renewal, suggesting that it may influence B-1 cell persistence and natural antibody structure. Young (4-wk-old) male and female mice were placed on a fasting regimen (24-h fast weekly) or fed ad libitum for 12 wk. Here, we show that intermittent fasting modulates the antigen-specific natural IgM repertoire of young male and female mice, altering both peritoneal and splenic phosphatidylcholine-specific CD5+ B-1 cell repertoires, with the most pronounced effects in the spleen. Intermittent fasting increased the prevalence of germline-like (few N-additions) antibodies and led to an increase in V_H_11 usage, a variable gene mainly utilized during fetal development. Fasting also affected components of the serum repertoire; however, these effects differed between male and female mice. Female mice showed a significant decrease in phosphorylcholine- and pneumococcal polysaccharide serotype 3–specific IgM levels, whereas male mice showed a significant increase in phosphatidylcholine-specific IgM levels. We also observed a significant decrease in splenic CD5+ B-1 cells and serum interleukin-5 levels after fasting. These results suggest that intermittent fasting may help preserve or restore protective NAbs.

## Introduction

Natural antibodies (NAbs) are present prior to encountering exogenous antigen^1^ and play a vital role in maintaining immune homeostasis. In mice, these antibodies have been shown to be crucial for B cell development,^2–4^ repertoire selection,^3^^,^^5^ and IgG responses, both T cell independent and dependent.^4^^,^^6–9^ Furthermore, natural IgM is essential for controlling infections,^10–14^ clearing apoptotic cells,^15^ and binding oxidized low-density lipoprotein to mitigate inflammation in atherosclerosis.^16^^,^^17^ About 80% to 90% of NAbs are produced by CD5+ B-1 cells,^18^ which generate NAbs that provide serological protection against pneumococcal infection and sepsis by expressing a B cell receptor (BCR) repertoire with a germline-like structure that recognizes phosphatidylcholine (PtC)^19^ and phosphorylcholine (PC).^20^ The prototypical CD5+ B-1 anti-PC Ab, T15, lacks N-additions and is highly protective against *Streptococcus pneumoniae* infection.^21^^,^^22^ Approximately 5% to 15% of peritoneal CD5+ B-1 cells are specific for PtC, an antigen found on senescent red blood cells as well as on bacterial cell membranes.^23^^,^^24^ Anti-PtC antibodies have been shown to be essential in protection from bacterial sepsis.^25^

This structure of protective natural antibodies is termed germline-like because of the minimal insertion of nontemplated nucleotides (N-additions) and limited somatic hypermutation in the CDR-H3 region of the BCR.^26–28^ During V(D)J recombination of the BCR, N-additions can be inserted into the V-D and/or D-J junctions by the enzyme terminal deoxynucleotidyl transferase (TdT).^29^ In mice, TdT is not expressed during fetal life,^30^ which is the main developmental period for CD5+ B-1 cells; therefore, fetal development allows for germline-like antibodies (lacking N-additions) to develop.^31^ Self-renewal of these fetal-derived CD5+ B-1 cells enables the production of germline-like antibodies in adulthood. Overexpression of TdT during fetal development results in anti-PC antibodies that are not protective against *S. pneumoniae* infection,^32^ suggesting that N-addition can be detrimental to microbial protection; thus, structure is important for the protection provided by evolutionarily conserved natural antibodies.

We have recently shown that the protective capacity of natural IgM against *S. pneumoniae* infection is maintained in aged female mice but decreases in aged male mice.^33^^,^^34^ This loss of protection was not accounted for by changes in serum IgM levels. Rather, we observed an increase in N-additions in the cellular IgM repertoire of both PC- and PtC-specific CD5+ B-1 cells from aged male mice compared with those from aged female mice.^33^^,^^34^ In aged female mice, CD5+ B-1 cells continue to produce protective, germline-like IgM (few N-additions), whereas aged male mice have CD5+ B-1 cells that produce less protective, non–germline-like IgM (many N-additions). These previous results identified a specific age- and sex-related defect in the innate barrier (natural IgM produced by CD5+ B-1 cells) against *S. pneumoniae* in aged males, whereas aged females retained this protection. This led us to examine strategies to preserve the protective capacity of CD5+ B-1 cell–derived natural antibodies.

B-1 cell development occurs in waves from multiple precursors, starting in the yolk sac of mice throughout fetal life and persisting into adulthood, mainly via the ability of B-1 cells to self-renew.^35–37^ Autophagy has been implicated in CD5+ B-1 cell maintenance, as loss of autophagy related genes such as Atg7 reduces B-1 cell self-renewal.^38^ Intermittent fasting has been shown to increase stem cell self-renewal^39^^,^^40^ and activate autophagy.^41–43^ These previously published studies provided an initial rational to investigate whether intermittent fasting could influence the natural IgM repertoire of young male and female antigen-specific CD5+ B-1 cells. We focused specifically on PtC-specific B-1 cells, a subset comprising 5% to 15% of B-1 cells that provide robust protection against sepsis, as well as potential cross-protection against pathogens such *as S. pneumoniae.*^23–25^^,^^44^ Importantly, a few studies have shown changes in PtC-specific CD5+ B-1 cells in metabolically altered environments. CD5+ B-1 cells obtained from lean healthy mice preferentially phagocytosed beads coated with PtC-coated compared with non–PtC-coated beads; however, this preferential phagocytosis was lost in obese mice fed a high-fat diet.^45^ This loss of phagocytic specificity for PtC in obesity has implications for the maintenance of tissue homeostasis. Furthermore, in a previous collaborative study, we demonstrated alterations in the CD5+ B-1 cell NAb repertoire toward a PtC specificity in mice with hyperlipidemia.^46^ Because these studies demonstrated that metabolic changes influence PtC-specific CD5+ B-1 cells, we examined the PtC-specific CD5+ B-1 cell repertoire in mice subjected to intermittent fasting. We discovered that intermittent fasting affects both peritoneal and splenic PtC-specific CD5+ B-1 cell repertoires in male and female mice. Interestingly, the effects were more pronounced in the splenic compartment than in the peritoneal cavity (PerC). These findings suggest that intermittent fasting may help maintain or increase protective germline-like natural antibodies in males. These results in young mice offer a promising potential avenue for preserving or augmenting protective natural antibodies in old age.

## Methods

### Mice

Male and female BALB/cByJ mice were obtained from the Jackson Laboratory at 3 wk of age and were housed in our vivarium until they reached the indicated age. For each independent experiment, mice were age matched (same date of birth), purchased at the same time, and maintained under identical conditions before assignment to fasting or control groups. The mice were housed at 5 mice per cage with a 12-h light/dark cycle and ad libitum access to water. Food was restricted at the times described in the fasting protocol below. Both male and female mice were used in this study. Mice were cared for and handled in accordance with the Guide for the Care and Use of Laboratory Animals (National Institutes of Health) and the institutional guidelines. All animal studies were approved by the Western Michigan University Homer Stryker M.D. School of Medicine Institutional Animal Care and Use Committee.

### Intermittent fasting protocol

Young (4-wk-old) male and female mice were placed on a fasting regimen or fed ad libitum for 12 wk. The fasting regimen consisted of removing food for one 24-h period each week for 12 wk. On the day food was removed, the mice were weighed and placed in a clean cage. The same procedure was performed for the group that was not fasted and fed ad libitum. All mice had ad libitum access to water. After 24 h without food, the food was replaced and removed again the following week (removed on Monday, replaced on Tuesday, and removed again the following Monday). This protocol was repeated for 12 wk, at which time the mice were euthanized, and tissues were collected at the end of the final 24-h fast.

### Cell purification and flow cytometry

Peritoneal lavage and spleen removals were performed on euthanized mice. Spleens were homogenized using the Miltenyi gentleMACS dissociator and then passed through a 70-µm cell strainer. All samples were treated with red blood cell lysis buffer for 2 min (Lonza), subsequently diluted with Hanks’ Buffered Salt Solution with 2.5% fetal bovine serum, and then centrifuged at 1,200 rpm for 10 min. The cells were resuspended in Hanks’ Buffered Salt Solution with 2.5% fetal bovine serum, stained with immunofluorescent antibodies, and analyzed using an Influx cell sorter (BD Biosciences) with gating on live cells by forward side scatter and/or Aqua Live/Dead stain (Invitrogen). Cells from 5 mice per group were pooled prior to single-cell sorting. Images were constructed using FlowJo 10.0 software (BD Biosciences) and OMIQ (Dotmatics). The following antibodies were obtained from BD Pharmingen: CD19 (clone ID3), CD43 (clone S7), B220/CD45 (clone RA3-6B2), CD23 (clone B3B4), and CD5 (clone 53-7.3). FITC-labeled PtC liposomes (purchased from Dr. Aaron Kantor), diluted at 1:30,000, were used to detect PtC+ B cells, as previously described.^47^ The composition of the PtC liposomes used is DSPC:DSPG:Chol (45:5:50 molar ratio).

### Single-cell sequencing and analysis

PtC+CD5+ B-1 cell populations were single-cell sorted using an Influx cell sorter (BD Biosciences) into a 96-well plate containing lysis buffer (RNaseOut, 5× Buffer, DTT, IgePAL, carrier RNA; Invitrogen). Postsort reanalysis of CD5+ B-1 cell populations showed them to be ≥98% pure. To obtain cDNA, a 20 µL reverse transcription reaction was run per well using the SuperScript III enzyme and random hexamers (Invitrogen). Qiagen’s HotStart Taq Plus master mix kit was used to perform the first round of polymerase chain reaction (25 µL reaction) using 2.5 µL of complementary DNA diluted 1:2 and the following primers: MsVHE and MsCµE each at 0.6 µM, as previously described.^28^^,^^47^ Each 25 µL reaction was run as follows: 95 °C for 5 min; 35 cycles at 94 °C for 30 s, 50 °C for 30 s, and 72 °C for 30 s, and then a final extension at 72 °C for 10 min. The product from this first reaction was then diluted 1:100 in dH_2_O and 2 µL was used in the second semi-nested 25 µL reaction using the following primers: MsVHE and MsCµN each at 0.6 µM, as previously described.^28^^,^^47^ The second reaction was run as follows: 95 °C for 5 min; 40 cycles at 94 °C for 30 s, 53 °C for 30 s, and 72 °C for 30 s, and then a final extension at 72 °C for 10 min. The products were run on a Qiaxcel (Qiagen). Polymerase chain reaction products were sequenced (Genewiz, Azenta) using MsVHE primers. Sequences were analyzed using the online sequence analysis tool, IMGT/HighV-Quest.^48^

### Total immunoglobulin and cytokine serum analysis

Serum was obtained from mice fed ad libitum and fasted as described within the intermittent fasting protocol section. Serum samples were analyzed for total IgM or IgG using ELISA, according to the manufacturer’s instructions (Bethyl Laboratories). Cytokines, including interferon γ (IFN-γ), interleukin (IL)-10, IL-1β, IL-4, IL-5, KC/GRO, and tumor necrosis factor α, as well as active glucagon-like peptide-1 and glucagon, were quantified using a chemiluminescence-based multiplex assay (Meso Scale Discovery V-Plex) on a QuickPlex SQ 120 instrument. Data were analyzed using DISCOVERY WORKBENCH 5.1 software (Meso Scale Discovery ) and GraphPad Prism (version 10.6.1; GraphPad Software). Only measurements with a calculated coefficient of variation <10% were included in the analyses.

### DOPC, PC, and pneumococcal polysaccharide serotype 3 serum ELISA analysis

Serum was obtained from mice fed ad libitum and fasted as described within the intermittent fasting protocol section. Serum samples were analyzed for antibodies against DOPC using ELISA. ELISA strips were obtained from Avanti Polar Lipids and precoated with DOPC. Wells were blocked with 200ul of 3% fatty acid free bovine serum albumin in phosphate-buffered saline (PBS) for 1 h at room temperature with gentle shaking and then washed 3 times with 1× PBS. Diluted serum was added at 50 µL per well and incubated for 1 h at room temperature with gentle shaking. The wells were then washed 3 times with 1× PBS. Bound antibody was measured using HRP-conjugated goat anti-mouse IgM (Bethyl Labs) at 1:20,000. NC-17, kindly provided by Dr. Gregg Silverman, was used as a standard and included on each plate. IgM-specific PC and pneumococcal polysaccharide serotype 3 (PPS3) serum levels were measured by coating 96-well plates with PC-BSA (Biosearch Technologies) or PPS3 (American Type Culture Collection) at 5 µg/mL in 1× PBS, as previously described.^34^^,^^49^ IgM standards were included on each plate and PC/PPS3-specific antibody levels were interpreted as volume equivalent of the IgM standard, as described in Shriner et al.^49^ Therefore, the amount of PC- or PPS3-specific IgM was calculated relative to the IgM standard.

### Serum concentration of PtC

Serum was obtained from mice fed ad libitum and fasted as described within the intermittent fasting protocol section. PtC concentration was quantified using a colorimetric assay (Cayman Chemical) according to the manufacturer’s instructions.

### Statistical analysis

Statistical analyses were performed using GraphPad Prism. All statistical analyses are indicated in the figure legends. An outlier test was performed on all datasets using Prism’s ROUT method for identifying outliers. Outliers were removed when detected using Prism’s ROUT method with the coefficient Q set at 1%.

## Results

### Intermittent fasting does not alter body weight in young male or female mice

**Figure 1:**
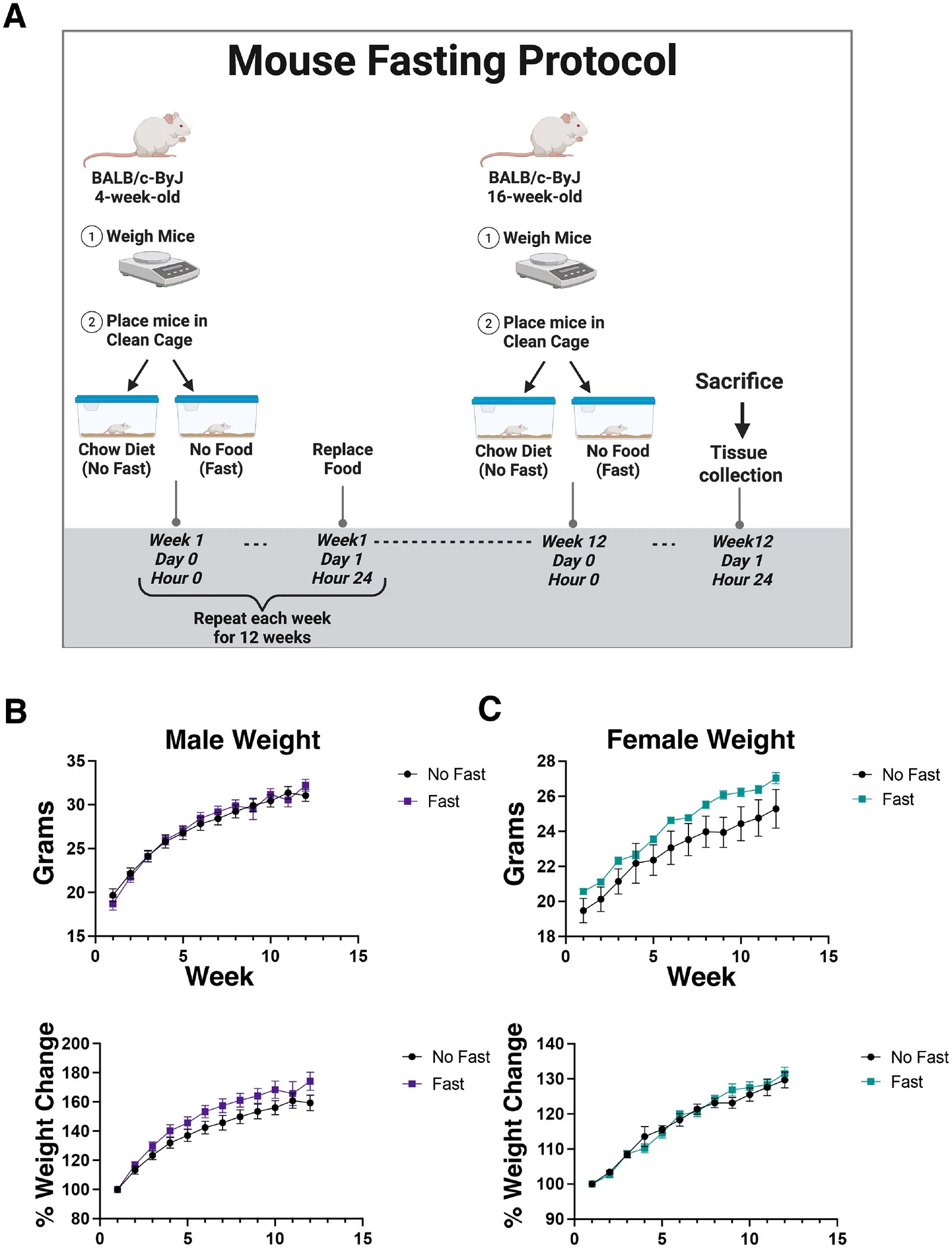
Mouse fasting protocol and weights over a 12-wk intermittent fasting period. (A) Fasting protocol. Created in Biorender. (B, C) Young (4-wk-old) male (B) and female (C) BALB/c-ByJ mice were fasted (as described in the fasting protocol) for 12 wk. Weights were measured once a week for each mouse. (Top) The weight measure in grams; (bottom) the percent weight change. Results based on 2 independent experiments for male mice (n = 10 mice total per group = nonfast vs. fast, 5 mice/experiment/group) and separately, 2 independent experiments for female mice (n = 10 mice total per group = nonfast vs. fast, 5 mice/experiment/group). Statistics used: 2-way repeated-measures analysis of variance with time as the with subjects’ factor and diet as the between-subjects factor. The Geisser-Greenhouse correction was applied when sphericity was violated. Post hoc comparisons were performed using Sidak’s multiple comparison tests. Data are presented as mean ± SEM.

Young male and female BALB-ByJ mice (4 wk old) were subjected to a 12-wk intermittent fasting regimen or were fed ad libitum (control group, no fast). The fasting protocol consisted of a single 24-h period without food each week, after which food was returned for the following 6 d. All mice were provided with water at all times. For both the fasting and ad libitum groups, the mice were transferred into a clean cage once per week at the time of food removal (food was removed only from the fasted group). At the end of the 24-h fast, the fasted mice remained in the same cage and were provided food for the following 6 d. Mice were weighed weekly at the time of food removal and bedding change (Fig. 1A).

There was no significant difference in either sex between the ad libitum controls (no fast) and fasted mice. Male mice showed no trend in weight change (Fig. 1B). Female mice exhibited a slight, nonsignificant trend of higher weights in the fasting group after week 6; however, this was not statistically significant (Fig. 1C). These results indicate that intermittent fasting under this protocol does not affect the body weight of male or female mice.

### Intermittent fasting induces sex-specific changes in the natural IgM repertoire of peritoneal PtC+ CD5+ B-1 cells

**Figure 2:**
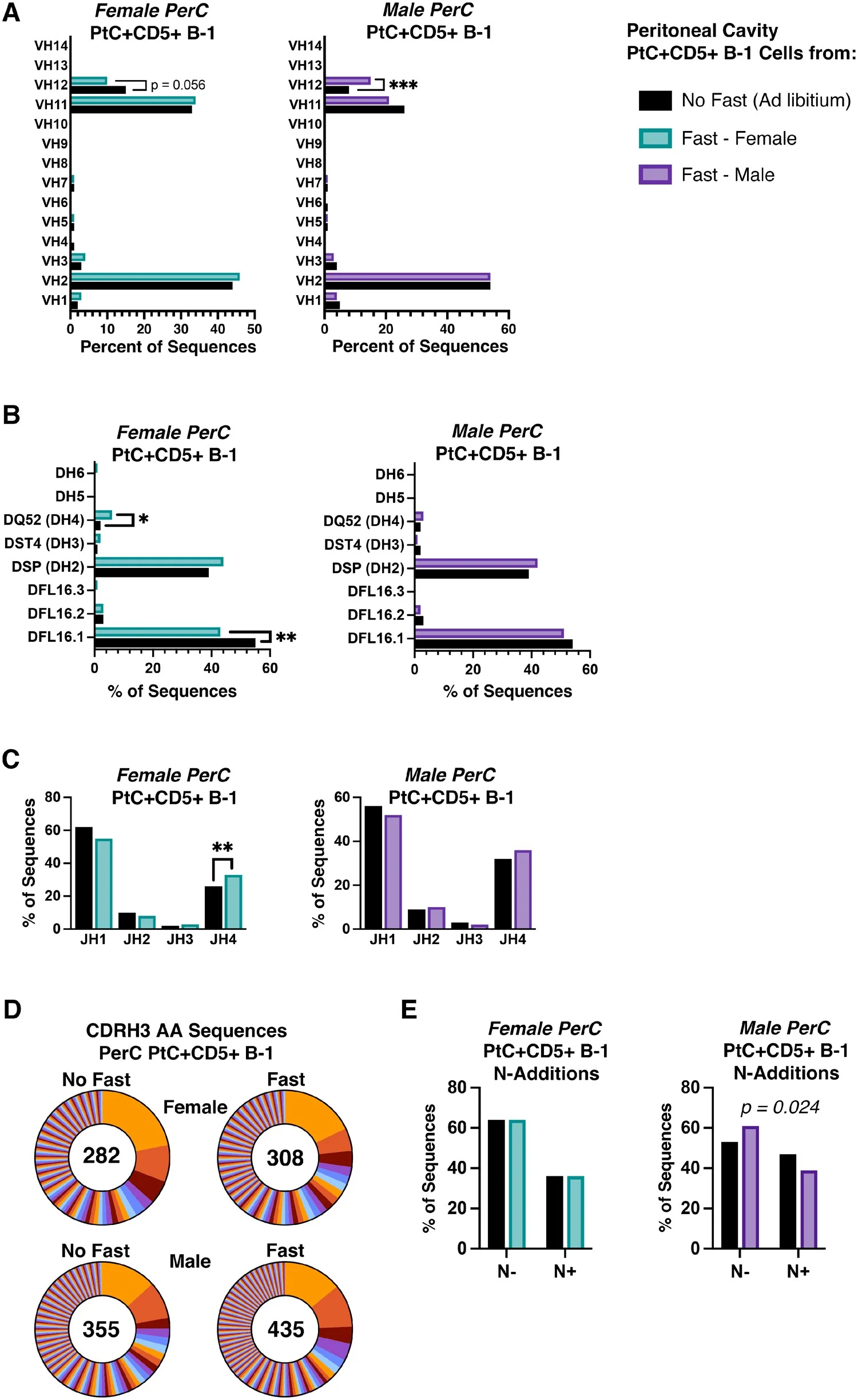
Repertoire analysis of peritoneal PtC-specific CD5+ B-1 cells in the absence or presence of intermittent fasting. Young (4-wk-old) male (graphs with purple bars) and female (graphs with teal bars) BALB/c-ByJ mice were fasted (as described in Fig. 1 and Methods) for 12 wk. Peritoneal washout cells were harvested from the euthanized mice. Single-cell suspensions were made and red blood cell lysed, and then PtC-specific CD5+ B-1 cells (B220^lo^, CD5^+^, CD43^+^, CD19^+^, CD23^−^, PtC^+^) were single-cell sorted into a 96-well plate with cell lysis buffer. The V_H_ region was amplified and sequenced. (A) The percent of V_H_ gene segment usage. (B) The percent of D_H_ gene segment usage. (C) The percent of J_H_ gene usage. (D) Distribution of CDR-H3 sequences (the number in the middle represents the total number of sequences). Each colored segment represents a unique CDR-H3 amino acid sequence. Larger segments reflect replicate sequences. (E) The number of N-additions at both junctions. Results based on 3 independent experiments for male mice (n = 15 mice total per group = nonfast vs. fast, 5 mice/experiment/group), and separately, 2 independent experiments for female mice (n = 10 mice total per group = nonfast vs. fast, 5 mice/experiment/group). Statistics used: 2 × 2 chi-square test. *P < 0.05, **P < 0.01, ***P < 0.001.

Following the 12-wk fasting protocol, PerC PtC-specific CD5+ B-1 cells (B220^lo^CD5^+^CD43^+^CD23^−^CD19^+^PtC^+^) were obtained by single-cell sorting to examine their repertoire. The sorting strategy is illustrated in the representative plots in Fig. S1A. The heavy chain of these single cells was examined for variable (V_H_), diversity (D_H_), and joining (J_H_) gene segment use, replicate CDR-H3 amino acid sequences, and germline status (Fig. 2).

Fasting resulted in significant differences in the use of V_H_, D_H_, and J_H_ gene segments in both males and females; however, these differences were sex specific. In PtC+ CD5+ B-1 cells from the PerC of female mice, fasting decreased V_H_12 usage (15% nonfasting vs. 10% fasting; *P* = 0.056) (Fig. 2A), increased D_H_ DQ52 (D_H_4) (2% nonfasting vs. 6% fasting; *P *= 0.0045), decreased DFL16.1 (55% nonfasting vs. 43% fasting; *P *= 0.003) (Fig. 2B), and significantly increased J_H_4 usage (26% nonfasting vs. 33% fasting; *P *= 0.0451) (Fig. 2C). In contrast, in male PerC PtC+ CD5+ B-1 cells, fasting significantly increased V_H_12 usage (8% nonfasting vs. 15% fasting; *P *= 0.0008) but caused no significant changes in the D_H_ or J_H_ segments (Fig. 2A–C).

The shifts observed in peritoneal V_H_ use were reflected in the replicate CDR-H3 amino acid sequences. Replicate sequences were defined as having identical CDR-H3 (same V_H_, D_H_, J_H_, N-additions, and P-insertions). We refer to these as replicates rather than clones because we have previously observed diversity in light chain use among such replicates.^47^ Replicate sequences were present in both male and female PerC PtC+ CD5+ B-1 cells under fasted and nonfasted conditions. Consistent with prior reports, a dominant CDR-H3 clonotype, MRYGNYWYFDV (V_H_11/D_H_1-1/J_H_1), associated with PtC binding, was observed in all groups. Importantly, this CDR-H3 was shown to cross-react with PC.^44^ The changes in male V_H_12 usage identified in Fig. 2A were reflected in the composition of the dominant replicate CDR-H3 sequences shown in Fig. S2B. Of the 282 sequences in nonfasted females, the top 3 replicates observed were MRYGNYWYFDV (22%; V_H_11/D_H_1-1/J_H_1), AGDNYGYWYFDV (9%; V_H_12/D_H_1-1/J_H_1), and ARDYYGSSYWYFD (6%; V_H_2-9/D_H_1-1/J_H_1). Of the 308 sequences in fasted females, the top 3 replicates were MRYGNYWYFDV (18%; V_H_11/D_H_1-1/J_H_1), ARAYYRYDYYAMDY (6%; V_H_2-9/D_H_2-14/J_H_4), and ADRDYYGSSYWYFDV (3%; V_H_2-9/D_H_1-1/J_H_1). In nonfasted males, of the 355 sequences, the top 3 replicates were MRYGNYWYFDV (13%; V_H_11/D_H_1-1/J_H_1), ARDYYGSSYWYFDV (9%; V_H_2-9/D_H_1-1/J_H_1), and MRYGGYWFDV (3%; V_H_11/D_H_1-1/J_H_1). In fasted males, of the 435 sequences, the top 3 replicates were MRYGNYWYFDV (14%; V_H_11/D_H_1-1/J_H_1), ARDYYGSSYWYFDV (10%; V_H_2-9/D_H_1-1/J_H_1), and AGDYDGYWYFDV (4%; V_H_12-3/D_H_2-3/J_H_1). These results, summarized in Figs. S2A and 2B, demonstrate that fasting shifts the use of CDR-H3 specificities in both male and female PtC+ CD5+ B-1 cells.

Importantly, fasting significantly increased the proportion of sequences lacking N-additions in male PtC+ CD5+ B-1 cell IgM (53% nonfasting vs. 61% fasting; *P *= 0.024) (Fig. 2E), indicating an enrichment of germline-like B-1 cells. In contrast, female PtC+ CD5+ B-1 cell IgM showed no such change in N-additions (N-additions in nonfasted females: 64%; N-additions in fasted females: 64%) after intermittent fasting. Thus, these data reveal a sex-specific effect of intermittent fasting on peritoneal antigen-specific CD5+ B-1 cells in mice. Both males and females exhibit modest reshaping of V_H_, D_H_, and J_H_ segment usage; however, only males selectively expand the germline-like B-1 cells.

### Intermittent fasting induces sex-specific changes in the natural IgM repertoire of splenic PtC+ CD5+ B-1 cells

**Figure 3:**
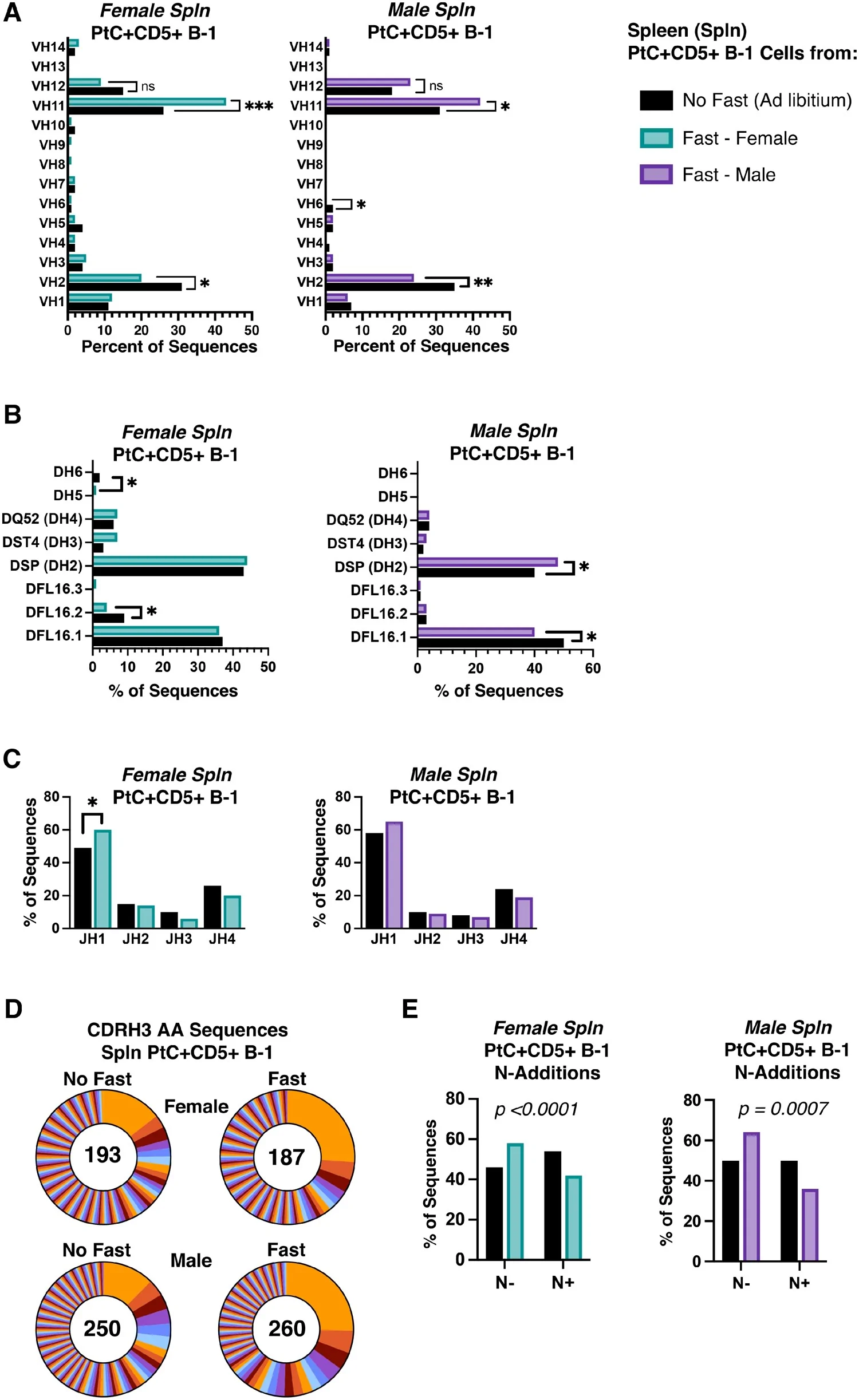
Repertoire analysis of splenic PtC-specific CD5+ B-1 cells in the absence or presence of intermittent fasting. Young (4-wk-old) male (graphs with purple bars) and female (graphs with teal bars) BALB/c-ByJ mice were fasted (as described in Fig. 1 and Methods) for 12 wk. Spleens were harvested from the euthanized mice. Single-cell suspensions were made and red blood cell lysed, and then PtC-specific CD5+ B-1 cells (B220^lo^, CD5^+^, CD43^+^, CD19^+^, CD23^−^, PtC^+^) were single-cell sorted into a 96-well plate with cell lysis buffer. The V_H_ region was amplified and sequenced. (A) The percent of V_H_ gene segment usage. (B) The percent of D_H_ gene segment usage. (C) The percent of J_H_ gene usage. (D) Distribution of CDR-H3 sequences (the number in the middle represents the total number of sequences). Each colored segment represents a unique CDR-H3 amino acid sequence. Larger segments reflect replicate sequences. (E) The number of N-additions at both junctions. Results based on 3 independent experiments for male mice (n = 15 mice total per group = nonfast vs. fast, 5 mice/experiment/group), and separately, 2 independent experiments for female mice (n = 10 mice total per group = nonfast vs. fast, 5 mice/experiment/group). Statistics used: 2 × 2 chi-square test. *P < 0.05, **P < 0.01, ***P < 0.001. ns, not significant.

To assess the impact of intermittent fasting on the splenic PtC+ CD5+ B-1 cell repertoire, we performed single-cell VDJ sequencing of sorted cells (B220^lo^CD5^+^CD43^+^CD23^−^CD19^+^PtC^+^) from the spleens of male and female mice following the 12-wk fasting protocol (Fig. 3). The sorting strategy is shown in the representative plots in Fig. S1B.

Fasting resulted in significant differences in the use of V_H_, D_H_, and J_H_ gene segments in both males and females. However, as in the PerC, some of these differences remained sex specific. Specifically, in PtC+ CD5+ B-1 cells from the spleens of female mice, fasting resulted in an increase in V_H_11 usage (26% nonfasting vs. 43% fasting; *P *< 0.0001) and a decrease in V_H_2 usage (31% nonfasting vs. 20% fasting; *P *= 0.0145) (Fig. 3A). Similarly, fasting males showed increased V_H_11 usage (31% vs. 42%; *P *= 0.0106), accompanied by decreases in both V_H_2 (35% vs. 24%, *P* = 0.0039) and V_H_6 usage (2% vs. 0%; *P *= 0.0121) (Fig. 3A). Analysis of the D_H_ gene segment usage revealed sex-specific effects. Postfast, females displayed decreased D_H_6 (2% nonfasting vs. 0% fasting; *P *= 0.049) and DFL16.2 frequencies (9% nonfasting vs. 4% fasting; *P *= 0.0302) (Fig. 3B). In contrast, fasted males showed increased DSP(D_H_2) usage (40% vs. 48%; *P *= 0.049) and decreased DFL16.1 frequency (50% vs. 40%; *P *= 0.0322) (Fig. 3B). Regarding J_H_ usage, females increased J_H_1 frequency postfast (49% nonfasting vs. 60% fasting; *P *= 0.028) and males showed no significant changes in J_H_ usage postfast (Fig. 3C).

Similar to the PerC, the shifts observed in splenic V_H_ use were reflected in the replicate CDR-H3 amino acid sequences (Fig. 3D). Both females and males increased the frequency of the replicate MRYGNYWYFDV (V_H_11/D_H_1-1/J_H_1) after fasting (Figs. S2C and S2D). This particular CDR-H3 amino acid sequence is known to bind PtC and is cross-reactive with PC.^44^ In nonfasted females, this CDR-H3 represented 15% of sequences, increasing to 26% in postfasting females. Males showed the same trend: MRYGNYWYFDV represented 12% of sequences in nonfasted males, rising to 25% after fasting (Figs. S2C and S2D).

Furthermore, analysis of N-additions demonstrated that fasting significantly increased the proportion of sequences lacking N-additions in both sexes (46% nonfasted vs. 58% fasted; *P *< 0.0001 in females; 50% nonfasted vs. 64% fasted; *P *= 0.0007 in males) (Fig. 3E). Together, these data suggest an enrichment of germline-like repertoires postfasting in both males and females. Overall, these findings indicate that intermittent fasting alters the repertoire of PtC+ CD5+ B-1 cells in a sex-dependent manner.

For single-cell sorting, B-1 cells from 5 mice per group were pooled prior to single-cell sorting in each experiment. Thus, individual mice were not sequenced separately. We acknowledge that this pooling strategy does not allow us to assess mouse-to-mouse variation within a given experiment. However, because these studies were performed in young, inbred mice purchased as cohorts with the same date of birth, we expect baseline repertoire variation between mice within each experiment to be limited. Importantly, the conclusions are based on independent fasting experiments rather than on a single pooled cohort: males were analyzed across 3 independent experiments and females across 2 independent experiments. We performed an additional per-experiment analysis of the splenic PtC+CD5+ B-1 cell V_H_ usage and N-addition analysis for both males and females (Fig. S3). Importantly, the per-experiment analysis demonstrates reproducibility of the trend across independent experiments.

### Intermittent fasting alters CDR-H3 hydrophobicity and amino acid use in PtC+ CD5+ B-1 cells

**Figure 4:**
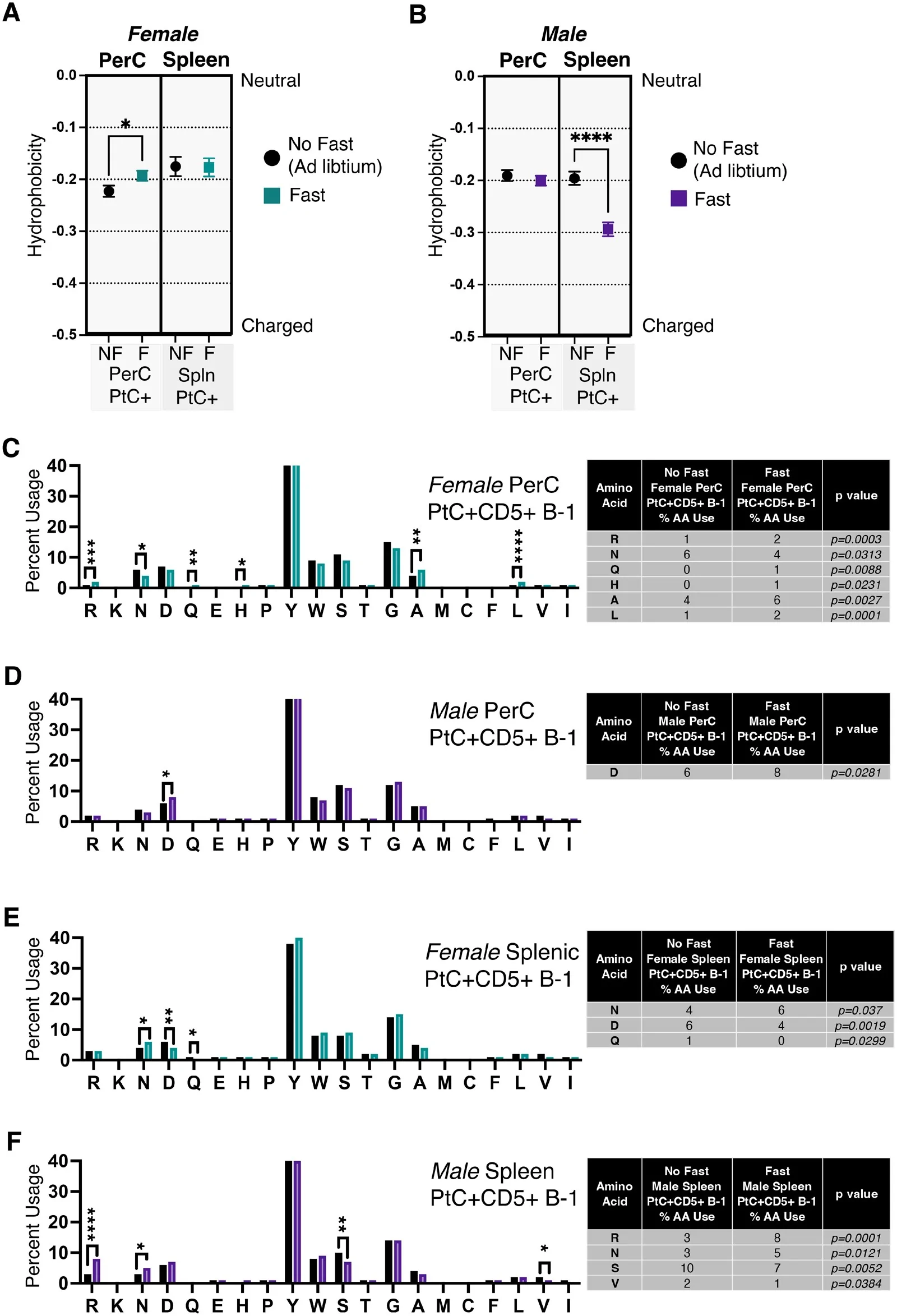
CDR-H3 hydrophobicity and amino acid changes with intermittent fasting. Peritoneal or splenic PtC+ CD5+ B-1 cells were single-cell sorted from female BALB/c-ByJ mice, as indicated in Figs. 2 and 3. The V_H_ region was amplified and sequenced, as detailed in the Methods. (A, B) The average charge of the CDR-H3 loop region of IgM from peritoneal or splenic PtC+ CD5+ B-1 cells in female (A) or male (B) mice. **(C–F)** The percentage of each amino acid used within the CDR-H3 loop was determined for each CD5+ B-1 cell subset, as indicated. The results are based on sequences obtained from experiments performed in Figures 2 and 3 (see Figs. 2 and 3 for the number of animals and independent experiment numbers). Statistics used: Mann-Whitney test (A, B) and chi-square test (C–F). *P < 0.05, **P < 0.01, ***P < 0.001, ****P < 0.0001. F, fast; NF, no fast.

To further characterize the effects of intermittent fasting on the PtC+ CD5+ B-1 cell repertoire, we analyzed the hydrophobicity and amino acid composition of the CDR-H3 region, which is the primary site of antigen binding. We first evaluated the average hydrophobicity of each CDR-H3 loop using the Kyte-Doolittle scale. Hydrophobic CDR-H3 loops have been shown to be critical for certain broadly neutralizing antibodies against HIV,^50^ whereas antibodies with highly charged CDR-H3 loops tend to be autoreactive.^51^^,^^52^ In female peritoneal PtC+ CD5+ B-1 cells, fasting significantly increased CDR-H3 hydrophobicity from −0.22 to −0.18 (*P *= 0.011) (Fig. 4A), whereas female splenic PtC+ CD5+ B-1 cells showed no significant change (Fig. 4A). In contrast, male splenic PtC+ B-1 cells exhibited a pronounced decrease in hydrophobicity postfasting, with values shifting from −0.19 in nonfasted mice to −0.31 postfasting (*P *< 0.0001) (Fig. 4B), whereas male peritoneal PtC+ CD5+ B-1 cells showed no significant change.

Mature murine B cells display a predominance of tyrosine and glycine within the CDR-H3 region.^53^^,^^54^ Alterations in the amino acid content of this region can lead to reduced B cell development, diminished antibody production, and increased susceptibility to infection.^55–57^ Analysis of CDR-H3 amino acid usage revealed distinct effects based on sex and compartment. Although the use of tyrosine and glycine did not change after intermittent fasting, changes in other amino acids were observed. In female peritoneal PtC+ CD5+ B-1 cells, fasting resulted in an increased use of arginine, glutamine, histidine, alanine, and leucine, whereas asparagine levels decreased (Fig. 4C). Male peritoneal PtC+ CD5+ B-1 cells displayed an increase in aspartic acid following fasting (Fig. 4D). In the spleen, female PtC+ CD5+ B-1 cells exhibited a significant increase in asparagine and reductions in aspartic acid and glutamine postfasting (Fig. 4E). Splenic male PtC+ CD5+ B-1 cells demonstrated broader alterations in amino acid composition postfasting, including increases in arginine and asparagine and decreases in serine and valine (Fig. 4F). Collectively, these data suggest that intermittent fasting modifies V_H_/D_H_/J_H_ usage and N-additions (Figs. 2 and 3) and reshapes the properties and amino acid composition of the CDR-H3 region in PtC-specific CD5+ B-1 cells in a manner that is both sex and tissue compartment dependent.

### Intermittent fasting modulates splenic CD5+ B-1 cell numbers in a sex-dependent manner

**Figure 5:**
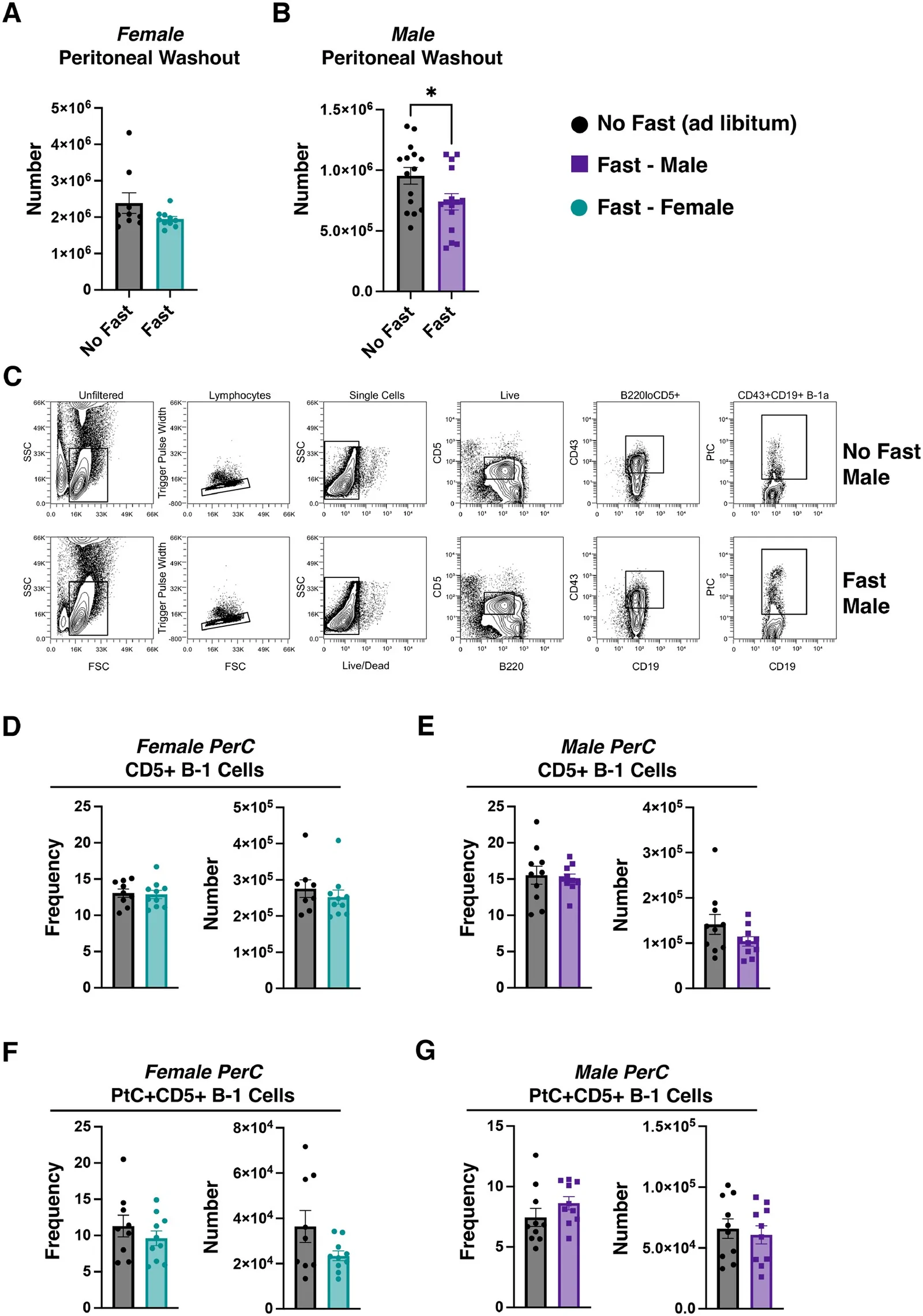
Number of PerC CD5+ B-1 cells does not differ between nonfasted and fasted mice. PerC CD5+ B-1 cells examined in young (4-mo-old) nonfasted and fasted male and female mice were assessed for their percentage and number. (A, B) Total number of PerC cells, (C) representative plots of PerC CD5+ B-1 cell gating, (D, E) percentage and total number of live peritoneal lymphocytes staining positive for CD5+ B-1 cells (B220^lo^CD5^+^CD19^hi^CD43^+^CD23^−^), and (F, G) percentage and total number of live peritoneal lymphocytes staining positive for PtC+CD5+ B-1 cells (B220^lo^CD5^+^CD19^hi^CD43^+^CD23^−^PtC^+^). Black circles represent either young male or female mice fed ad libitum/no fast, teal bars represent female fasted mice, and purple bars represent male fasted mice. Results based on 3 independent experiments for male mice (n = 15 mice total per group = nonfast vs. fast, 5 mice/experiment/group), and separately, 2 independent experiments for female mice (n = 10 mice total per group = nonfast vs. fast, 5 mice/experiment/group). Values are displayed as the mean ± SEM of individual mouse serum samples. Statistics used: Mann-Whitney *U* test. *P < 0.05. FSC, forward scatter; SSC, side scatter.

To determine whether intermittent fasting alters CD5+ B-1 cell populations, we assessed the number and frequency of CD5+ B-1 cells in the peritoneal (PerC) and splenic compartments. In the PerC, intermittent fasting caused a modest but significant reduction in the total number of PerC cells in males (Fig. 5B), but no change was observed in females after fasting (Fig. 5A). Fig. 5C illustrates the representative gating strategy for peritoneal CD5+ B-1 cells. In both females and males, no significant changes in the frequency or absolute number of CD5+ B-1 cells were observed (Fig. 5D, E). Similarly, the subset of peritoneal PtC+ CD5+ B-1 cells remained stable pre- and postfasting in both females (Fig. 5F) and males (Fig. 5G).

**Figure 6:**
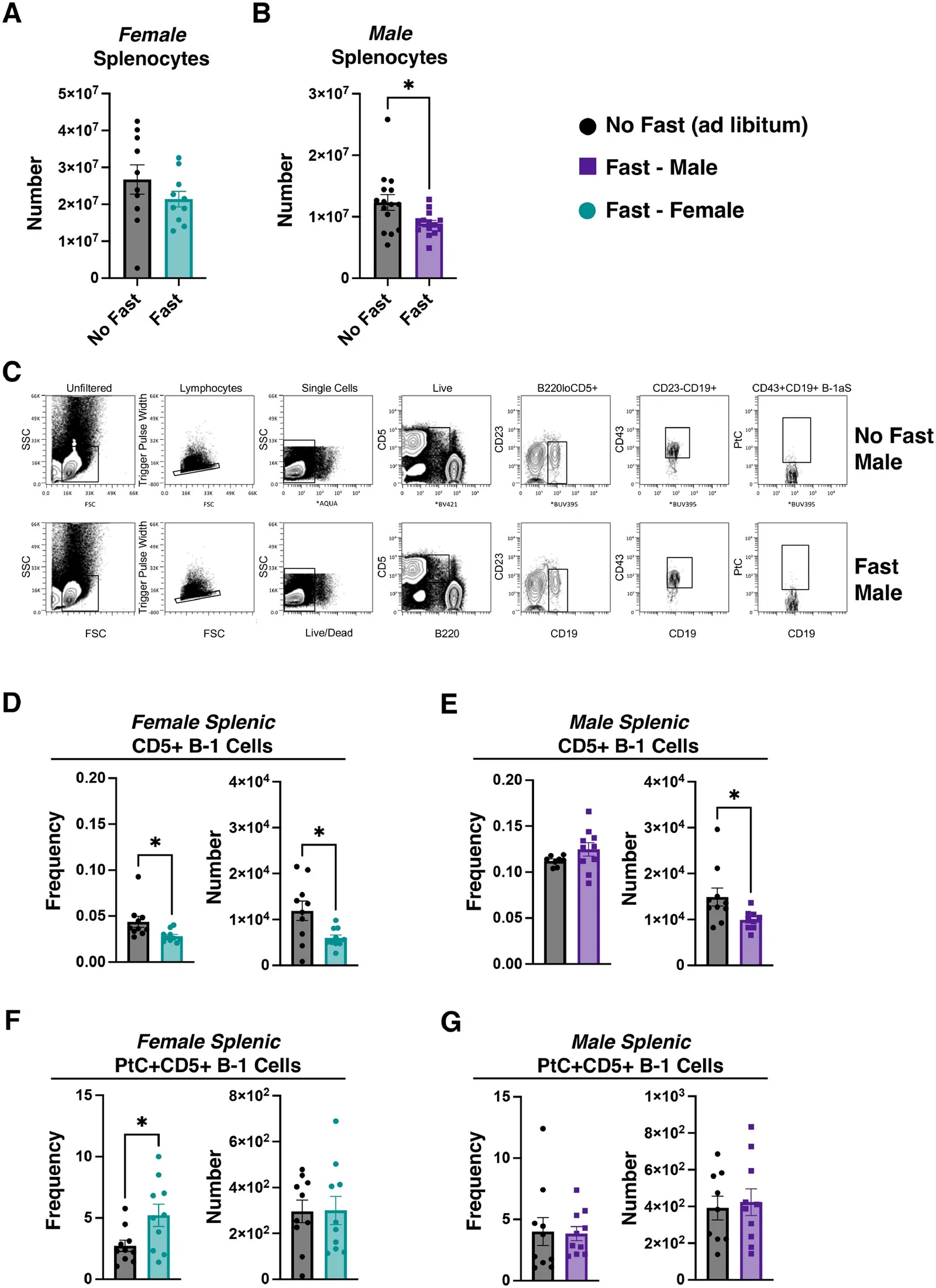
Number of splenic CD5+ B-1 cells in nonfasted and fasted mice. Splenic CD5+ B-1 cells examined in young (4-mo-old) nonfasted (ad libitum) and fasted male and female mice were assessed for their percentage and number. (A, B) Total number of splenocytes, (C) representative plots of splenic CD5+ B-1 cell gating, (D, E) percentage and total number of live splenocytes staining positive for CD5+ B-1 cells (B220^lo^CD5^+^CD19^hi^CD43^+^CD23^−^), and (F, G) percentage and total number of live splenocytes staining positive for PtC+CD5+ B-1 cells (B220^lo^CD5^+^CD19^hi^CD43^+^CD23^−^PtC^+^). Black circles represent either young male or female mice fed ad libitum/no fast, teal bars represent female fasted mice, and purple bars represent male fasted mice. Results based on 3 independent experiments for male mice (n = 15 mice total per group = nonfast vs. fast, 5 mice/experiment/group) and separately, 2 independent experiments for female mice (n = 10 mice total per group = nonfast vs. fast, 5 mice/experiment/group). Values are displayed as the mean ± SEM of individual mouse serum samples. Statistics used: Mann-Whitney *U* test. *P < 0.05. FSC, forward scatter; SSC, side scatter.

A different sex-dependent pattern emerged in the spleen. Male mice exhibited a significant decrease in total splenocytes postfast (Fig. 6B), whereas total splenocytes in females were unaffected (Fig. 6A). Figure 6C illustrates the representative gating strategy for splenic CD5+ B-1 cells. Splenic CD5+ B-1 cells in females demonstrated a significant reduction in both frequency and number postfasting (Fig. 6D), whereas males showed no change in frequency but a significant decrease in absolute number (Fig. 6E). Notably, the frequency of PtC+ CD5+ B-1 cells in females increased postfasting, although the total number remained constant (Fig. 6F), whereas males showed no changes in frequency or number (Fig. 6G). Collectively, these findings indicate that intermittent fasting affects CD5+ B-1 cell numbers in a sex- and niche-specific manner, with notable reductions in total PerC cells and splenocytes in males, decreased splenic CD5+ B-1 cells, and selective expansion of PtC+ CD5+ B-1 cell frequency in females.

### Intermittent fasting modulates serum antibody, cytokine, and PtC levels in a sex-dependent manner

**Figure 7:**
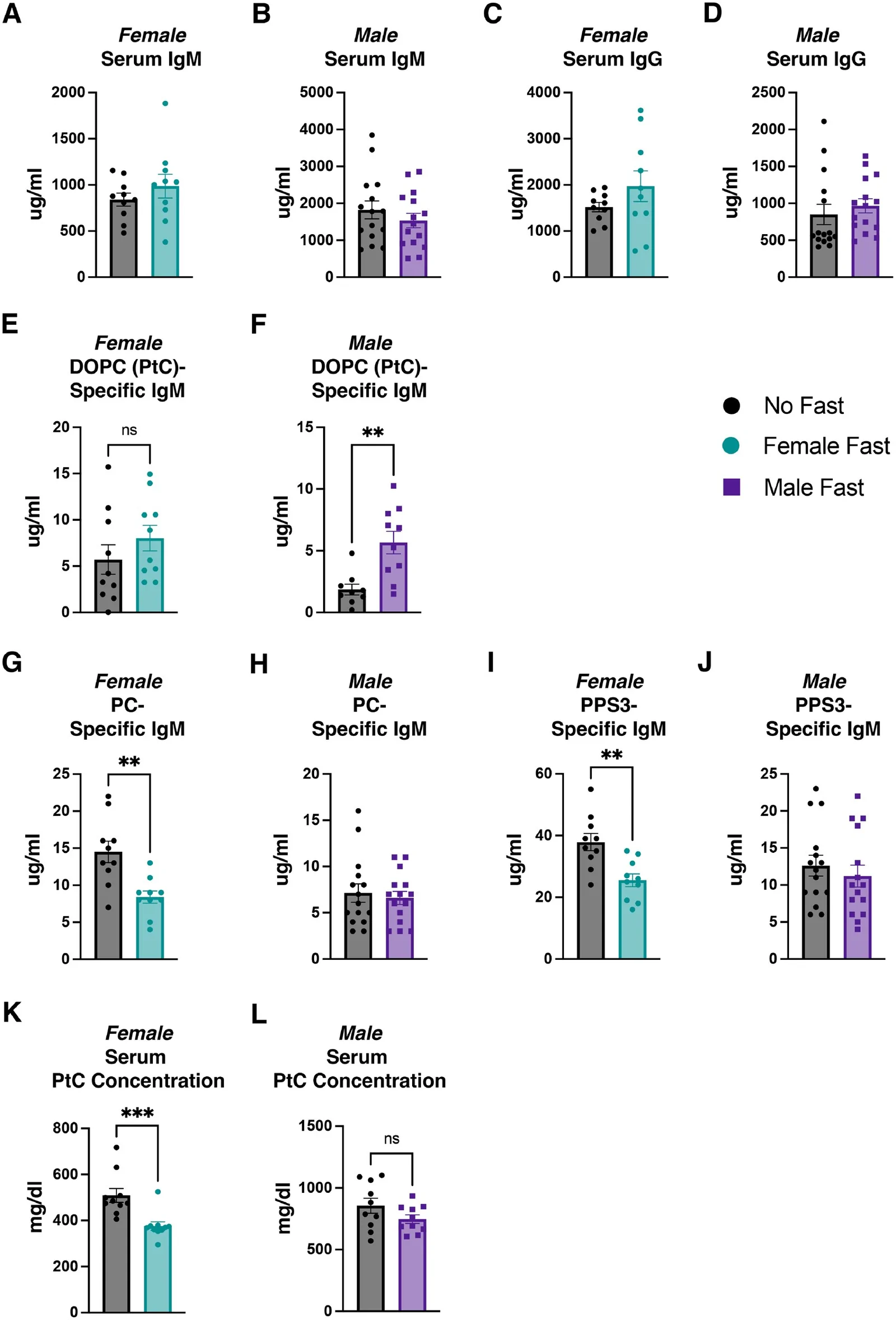
Serum IgM, IgG, antigen-specific IgM, and PtC concentration analysis in nonfasted and fasted male and female mice. Serum samples were obtained from nonfasted (ad libitum) or fasted male or female BALB/c-ByJ mice at the time of euthanasia and analyzed for (A, B) total IgM, (C, D) total IgG, (E, F) PtC-specific (DOPC-specific) IgM (G, H), PC-specific IgM, (I, J) PPS3-specific IgM, and (K, L) total serum PtC concentration. The amount of PtC-(DOPC)–specific IgM was calculated relative to a standard curve using the PtC-specific antibody NC-17. The amount of PC- and PPS3-specific IgM was calculated relative to an IgM standard curve. The results are displayed as the relative amount (calculated based on an IgM standard). Black circles represent either young male or female mice fed ad libitum/no fast, teal bars represent female fasted mice, and purple bars represent male fasted mice. Results based on 3 independent experiments for male mice (n = 15 mice total per group = nonfast vs. fast, 5 mice/experiment/group), and separately, 2 independent experiments for female mice (n = 10 mice total per group = nonfast vs. fast, 5 mice/experiment/group). Values are displayed as the mean ± SEM of individual mouse serum samples. Statistics used: Mann-Whitney test. **P < 0.01, ***P < 0.001. ns, not significant.

To assess the impact of intermittent fasting on antibody production, serum cytokines, and total serum PtC levels, we measured the following: total IgM, total IgG, PtC-specific IgM, PC-specific IgM, pneumococcal capsular PPS3-specific IgM, total PtC concentration, and cytokine levels in the serum of young male and female mice that were either fed ad libitum or fasted. The total serum IgM and IgG concentrations remained unchanged in both females (Fig. 7A, B) and males (Fig. 7C, D) after fasting. PtC-specific IgM levels were stable postfasting in females (Fig. 7E). In contrast, PtC-specific IgM levels significantly increased in males postfasting (1.9 ± 0.4 µg/mL nonfasted vs. 5.7 ± 0.9 µg/mL fasted; *P *= 0.003) (Fig. 7F). To further examine the actual natural antibody repertoire in the serum, we quantified the amount of PC and PPS3-specific IgM. After fasting, we found that both PC- and PPS3-specific IgM levels decreased in females and remained unchanged in males (Fig. 7G–J).

Interestingly, fasting has been shown to modulate PtC composition and metabolism.^58–61^ Here, we found that fasting led to a significant decrease in serum PtC concentration in females (509 ± 30 mg/dL nonfasted vs. 376 ± 18 mg/dL fasted; *P *= 0.0007) (Fig. 7K) and a nonsignificant decrease in males after fasting (855 ± 61 mg/dL nonfasted vs. 747 ± 35 mg/dL fasted; *P *= 0190) (Fig. 7L). These results demonstrate that fasting affects the serum PtC levels in mice.

**Figure 8:**
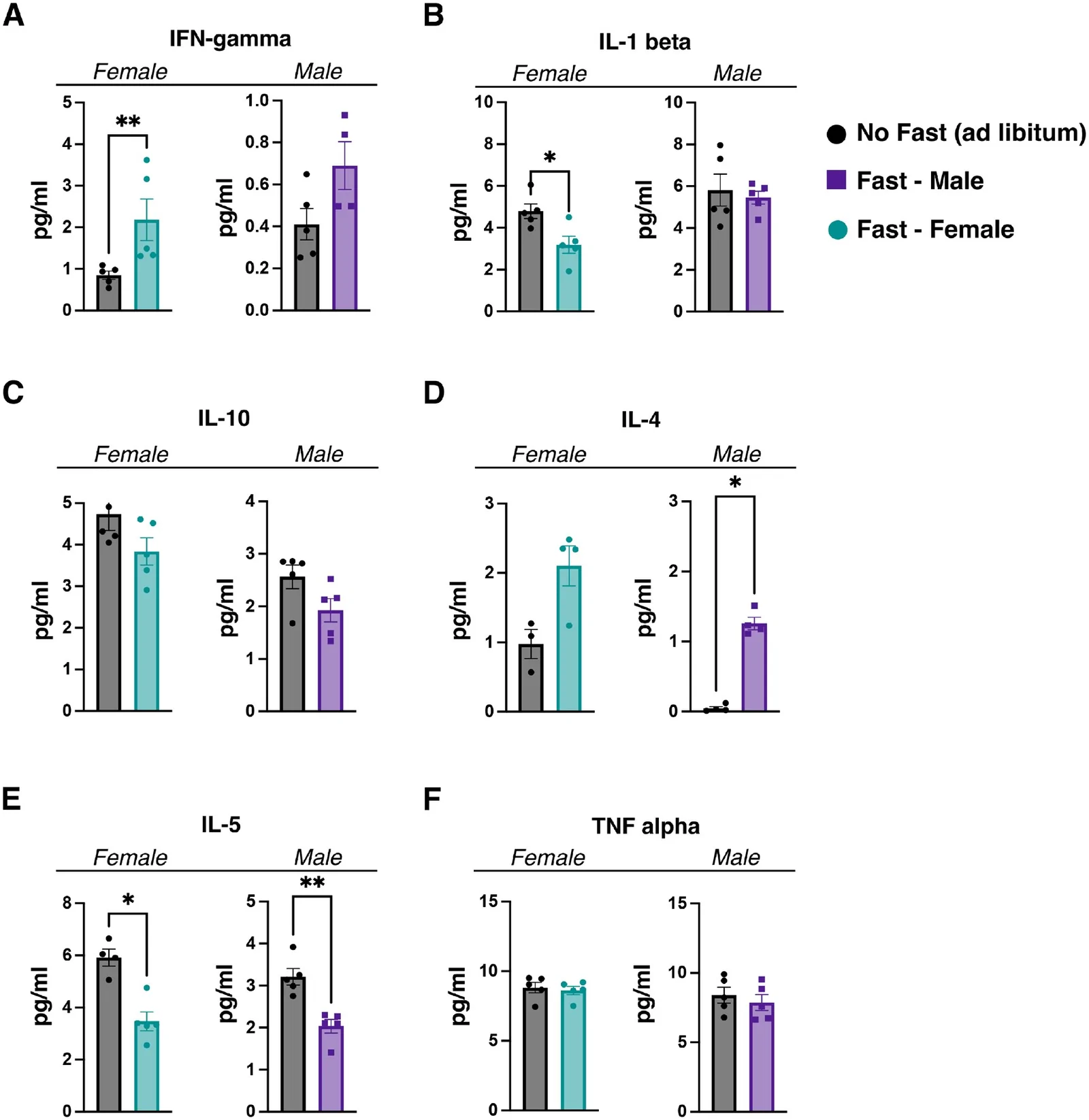
Serum cytokine analysis of nonfasted and fasted male and female mice. Serum samples were obtained from nonfasted (ad libitum) or fasted male or female BALB/c-ByJ mice at the time of euthanasia and analyzed for (A) IFN-γ, (B) IL-1β (C) IL-10 (D) IL-4, (E) IL-5, and (F) tumor necrosis factor (TNF) α. Black circles represent either young male or female mice fed ad libitum/no fast, teal bars represent female fasted mice, purple bars represent male fasted mice, and open black circles represent aged female mice. Results are based on 3 independent experiments for male mice (n = 15 mice total, 5 mice per experiment) and 2 independent experiments for female mice (n = 10 mice total, 5 mice per experiment). Serum samples from each experiment were pooled into 5 male and 5 female samples for evaluation of serum cytokine levels. Values are displayed as the mean ± SEM of pooled mouse serum samples. Statistics used: Mann-Whitney test. *P < 0.05, **P < 0.01.

Analysis of serum cytokines revealed sex-specific effects of fasting on cytokine levels. Female mice showed a significant increase in IFN-γ levels postfasting (0.85 ± 0.10 pg/mL nonfasted vs. 2.18 ± 0.50 pg/mL fasted; *P *= 0.0079) (Fig. 8A) and a modest but significant decrease in IL-1β (4.8 ± 0.34 pg/mL nonfasted vs. 3.2 ± 0.41 pg/mL fasted; *P *= 0.032) (Fig. 8B), while IFN-γ and IL-1β levels were unchanged in males postfasting (Fig. 8A, B). IL-10 levels remained unchanged in both sexes (Fig. 8C). Interestingly, males exhibited a significant increase in IL-4 postfasting (0.05 ± 0.002 pg/mL nonfasted vs. 1.3 ± 0.087 pg/mL fasted; *P *= 0.029), whereas females displayed a nonsignificant increase in IL-4 (Fig. 8D). Both sexes showed a significant decrease in IL-5 postfasting (males: 3.2 ± 0.19 pg/mL nonfast vs. 2.0 ± 0.16 pg/mL fasted; *P *= 0.0079; females: 5.9 ± 0.33 pg/mL nonfasted vs. 3.5 ± 0.36 pg/mL fasted; *P *= 0.016) (Fig. 8E). Tumor necrosis factor α levels remained unchanged in both males and females after fasting (Fig. 8F). Overall, these results indicate that intermittent fasting selectively and significantly alters PtC-specific IgM, PC-specific IgM, PPS3-specific IgM, serum PtC, and cytokine levels in a sex-dependent manner.

### Intermittent fasting modulates serum metabolic hormone levels in a sex-specific manner

**Figure 9:**
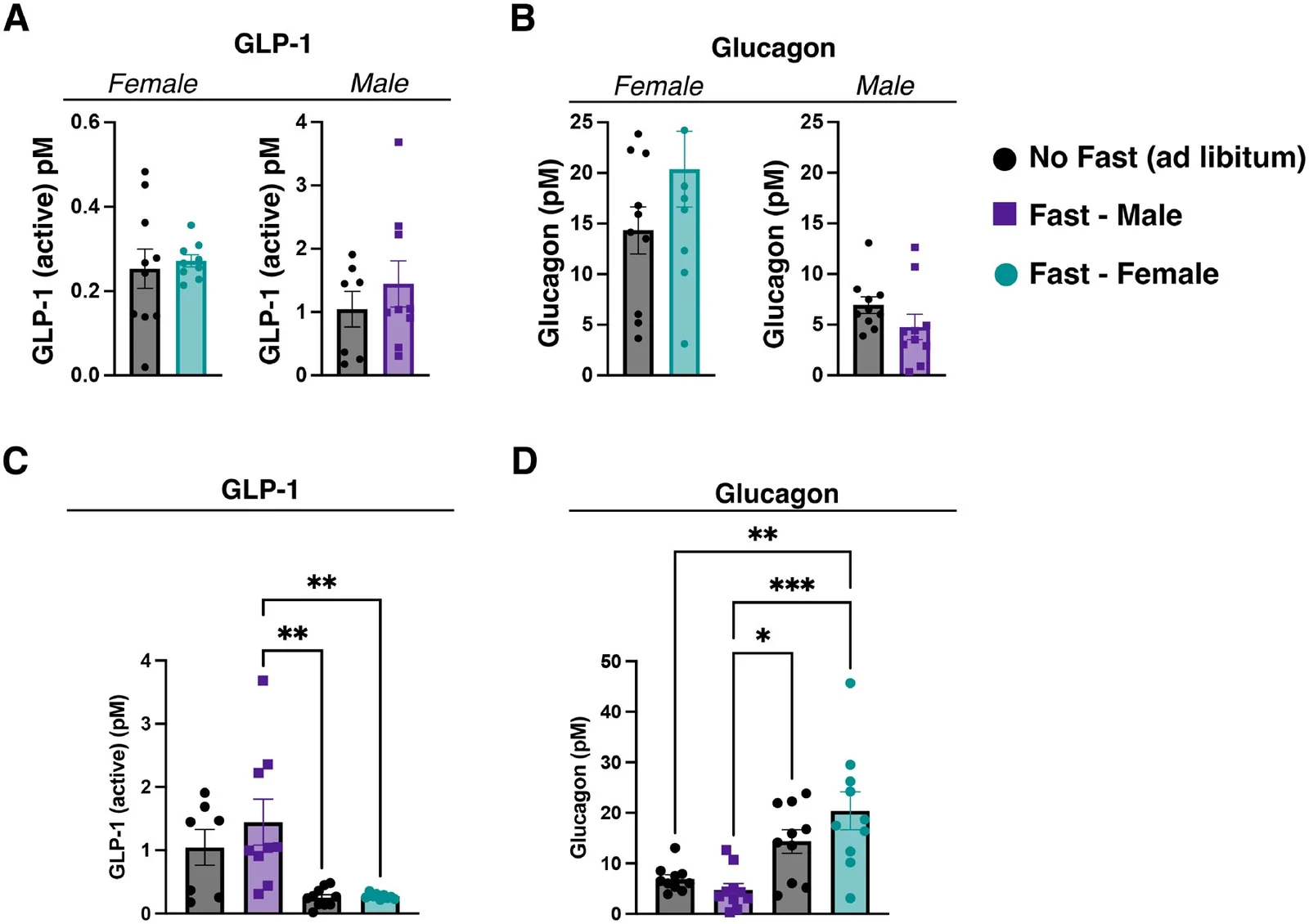
Serum metabolism hormones in nonfasted and fasted male and female mice. Serum samples were obtained from nonfasted (ad libitum) or fasted male or female BALB/c-ByJ mice at the time of euthanasia and analyzed for (A, C) active GLP-1 and (B, D) glucagon. Black circles represent individual mice. Dark gray bars indicate ad libitum, purple bars indicate fasted male mice, and teal bars indicate fasted female mice. (C, D) These are the same data displayed in panels A and B but arranged for male versus female comparison. Samples were run on MSD MESO Mouse Metabolism V-Plex plates, and the data were analyzed using Discovery Workbench. Only samples with coefficient of variation <10 were accepted as results. Results based on 2 independent experiments for male mice (n = 10 mice total, 5 mice per experiment) and 2 independent experiments or female mice (n = 10 mice total, 5 mice per experiment). Values are displayed as the mean ± SEM of individual mouse serum samples. Statistics used: analysis of variance with Tukey’s post hoc test. *P < 0.05, **P < 0.01, ***P < 0.001.

To assess whether intermittent fasting elicited sex-specific metabolic responses, the circulating levels of active glucagon-like peptide-1 (GLP-1) and glucagon were measured in the serum of male and female mice that were either fed ad libitum or fasted. Interestingly, we saw no significant differences in the level of active GLP-1 (Fig. 9A) or glucagon (Fig. 9B) after intermittent fasting in either males or females. However, we noticed males and females had significant differences in the level of these metabolic hormones. Under nonfasting conditions, no significant differences were observed between males and females in either active GLP-1 (1.05 ± 0.28 pM in males vs. 0.251 ± 0.04 pM in females) (Fig. 9C) or glucagon (6.94 ± 0.81 pM in males vs. 14.3 ± 2.32 pM in females) (Fig. 9D). In contrast, intermittent fasting revealed pronounced sex-dependent differences in the levels of both hormones. Fasted female mice exhibited significantly lower GLP-1 levels than fasted male mice (1.45 ± 0.36 pM in males vs. 0.27 ± 0.01 pM in females; *P *= 0.004) (Fig. 9C). Conversely, glucagon levels were significantly higher in fasted females (20.4 ± 3.75 pM) than in both fasted males (4.77 ± 1.24 pM; *P *= 0.0004) and nonfasted males (6.94 ± 0.82 pM; *P *= 0.0021) (Fig. 9D). These data suggest that intermittent fasting might elicit a sex-specific serum metabolic response that is not observed under ad libitum conditions; however, further studies are needed to explore this possibility.

## Discussion

Intermittent fasting has been extensively studied for its metabolic and health benefits, including modulation of body weight, insulin sensitivity, and overall metabolic homeostasis.^62–66^ However, outcomes can vary widely depending on the fasting regimen, duration, and subjects’ sex and age.^67–71^ In this study, young male and female BALB/c-ByJ mice were subjected to a weekly 24-h fast for 12 wk. Under this fasting protocol, all mice maintained stable body weights, with females showing only a slight, nonsignificant trend toward higher weights after week 6. These results indicate that this intermittent fasting protocol does not induce weight loss in young mice, aligning with prior reports that short-term or infrequent fasting has a minimal impact on body mass in healthy young rodents.^72^^,^^73^ The absence of significant changes in weight between males and females provides a stable baseline for investigating immunological effects, without the confounding influence of differential weight loss.

NAbs provide essential and immediate protection against bacterial and viral infection.^1^^,^^74^^,^^75^ The effectiveness of NAbs is attributed to their distinct characteristics (e.g. germline-like, few N-additions) resulting from the unique development of CD5+ B-1 cells, which generate 80% to 90% of NAbs.^74^^,^^75^ We have previously shown that aged male mice produce nonprotective, non–germline-like (many N-additions) natural IgM, whereas aged females retain protective, germline-like (few N-additions) natural IgM against pneumococcal infection.^33^^,^^34^ These results were shown for both PC- and PtC-specific CD5+ B-1 cells.^33^^,^^34^^,^^76^ We aimed to determine whether we could modulate the CD5+ B-1 cell–derived natural IgM cellular repertoire to preserve protective germline-like antibodies. One rationale for examining intermittent fasting was prior evidence has shown that fasting can enhance the self-renewal capacity of stem cells^39^^,^^40^ and increases autophagy.^41–43^ Because long-lived germline-like B-1 cells develop during early fetal life and persist into adulthood via self-renewal,^36^ which is dependent on autophagy,^38^ we hypothesized that intermittent fasting might influence the maintenance and repertoire composition of these early CD5+ B-1 cells, thereby preserving protective germline-like antibodies.

We specifically examined the effect of intermittent fasting on PtC-specific CD5+ B-1 cells, which make up 5% to 15% of CD5+ B-1 cells.^23^^,^^24^ Importantly, intermittent fasting has been shown to modulate PtC composition and metabolism,^58–61^ which we also observed herein with a decrease in serum PtC level. In addition, PtC-specific CD5+ B-1 cells have been shown to expand in healthy aged mice,^33^^,^^34^^,^^76^^,^^77^ aged hyperlipidemic mice,^46^ and mice with lupus-like disease.^78^ Our results demonstrate that a 24-h fast per week for 12 wk modulates the cellular/available PtC+CD5+ B-1 cell repertoire as well as the actual serum repertoire. Most changes in the cellular repertoire were observed in the splenic compartment. This is noteworthy because B-1 cells residing in the spleen are the primary source of circulating natural antibody.^79^^,^^80^ Remarkably, in both males and females, we found fewer N-additions (germline-like) in splenic PtC+CD5+ B-1 cell IgM from fasted mice. These findings demonstrate that intermittent fasting in young mice can alter the available cellular natural antibody repertoire. The germline status of PtC+ antibodies from fasted mice (very few N-additions) suggests that fasting may preserve and/or restore protective NAbs over time. Intermittent fasting also altered the serum repertoire; however, responses differed between males and females. Fasted males demonstrated significantly increased levels of serum PtC-specific IgM compared with nonfasted males, whereas the levels in females increased but not significantly. Conversely, females showed a significant decrease in both PC- and PPS3-specific IgM levels after fasting, whereas males remained unchanged. These findings demonstrate that both the cellular and actual natural antibody repertoires are impacted by intermittent fasting; however, the serum repertoire is further influenced by biological sex differences. The frequency and/or number of CD5+ B-1 cells available to produce NAbs could impact the quantity of NAbs present.

Fasting shifts nutrient use from glucose to fatty acids. Once glycogen is depleted, adipose tissue initiates lipolysis, releasing fatty acids into the bloodstream. CD5+ B-1 cells take up exogenous fatty acids and undergo apoptosis when fatty acid synthesis is inhibited.^38^ As fasting increases fatty acids^81^ and B-1 cells utilize and synthesize fatty acids, we hypothesized that CD5+ B-1 cell numbers might be impacted after fasting. Interestingly, we observed no changes in the number or frequency of peritoneal CD5+ B-1 cells. Conversely, in the spleen, we observed a significant decrease in splenic CD5+ B-1 cells in both male and female mice after fasting. Future studies are needed to investigate how changes in fatty acids after fasting might impact splenic B-1 cell numbers. However, the decrease in CD5+ B-1 cells aligns with the decrease in serum IL-5 levels observed in both males and females after fasting, as IL-5 is required for B-1 cell maintenance.^82^ This decrease in IL-5 is consistent with prior reports that fasting can suppress cytokines linked to eosinophil recruitment and B cell proliferation.^83^^,^^84^ These results suggest a possible increase in splenic B-1 cell turnover in response to intermittent fasting, as described for other immune cells.^84–89^ The increase in B-1 cell turnover could trigger self-renewal of the remaining B-1 cells to fill the B-1 cell niche, at which point selective pressures would shape the resulting repertoire. The increase in V_H_11 utilization observed in response to fasting suggests turnover and subsequent selection. Recombinations involving V_H_11 do not tolerate N-additions^90^; V_H_11 is therefore at a developmental disadvantage during adult life (when the enzyme TdT, which adds N-additions during VDJ recombination, is expressed). As a result, V_H_11 recombination occurs mostly during early life, when TdT is not present. Thus, the expansion of V_H_11 in adult mice after fasting suggests the selection of early fetal-derived B-1 cells utilizing V_H_11.

We previously demonstrated that selection affects the B-1 cell repertoire over time.^33^ Factors that influence the selection of the B-1 cell pool over time are still being elucidated; however, we have shown that biological sex differences play a role in shaping the repertoire over time.^34^^,^^76^ Previous reports have shown that male and female mice respond differently to metabolic interventions.^91^ Here, we found repertoire differences in V_H_, J_H_, and D_H_ usage; hydrophobicity of the CDR-H3 loop; and amino acid usage within the CDR-H3 loop between males and females. Furthermore, only females showed an increase in splenic PtC-specific CD5+ B-1 cells and a significant decrease in PC- and PPS3-specific serum IgM levels after fasting. These results suggest that intermittent fasting selectively preserves or expands antigen-specific cell subsets. One potential explanation for the fasting-associated repertoire remodeling observed in this study is altered exposure to endogenous ligands recognized by PtC- and PC-specific CD5+ B-1 cells. Although these natural antibodies are well known for their role in protection against bacterial pathogens, PtC and PC epitopes are also present on senescent erythrocytes, apoptotic cells, and oxidized lipoproteins. Natural IgM recognizing these structures contributes to tissue homeostasis and clearance of cellular debris.^15^^,^^16^^,^^92–94^ Thus, PtC-specific CD5+ B-1 cells are likely subjected to continual antigen-driven selection even in the absence of overt infection. Consistent with this possibility, we observed changes in circulating PtC levels following fasting. Previous studies have shown that intermittent fasting alters lipid metabolism and PtC composition in multiple tissues, including membrane phospholipid remodeling and changes in PtC species abundance.^60^^,^^95^^,^^96^ Therefore, alterations in lipid metabolism or the availability and presentation of endogenous PtC-containing ligands may contribute to the repertoire shifts observed after intermittent fasting.

In addition to immune-intrinsic changes, we observed sex-specific metabolic responses during intermittent fasting. We examined GLP-1, which promotes insulin secretion and reduces appetite, and glucagon, which raises blood glucose levels, together providing complementary regulation of energy and glucose homeostasis.^97^ Within males and females, intermitted fasting did not significantly alter circulating GLP-1 or glucagon levels. However, fasting revealed pronounced sex-dependent differences, with females exhibiting reduced active GLP-1 and elevated glucagon levels compared with males under fasting condition. Both GLP-1 and glucagon have been reported to influence immune cell metabolism, survival, and inflammation,^98–102^ suggesting that fasting-induced hormonal environments may influence the changes in B-1 cells reported here. Although our study did not directly test the hormonal mechanism, these associations suggest that sex-dependent metabolic adaptations to intermittent fasting may contribute to the changes observed in antigen-specific CD5+ B-1 cells. The differences we observed between males and females in response to intermittent fasting suggest that hormonal, metabolic, and antigenic differences between the sexes may dynamically influence the maintenance of the B-1 cell pool.

While our study is limited in that it provides only correlative clues to the mechanism, it begins to define the impact of metabolic interventions, such as intermittent fasting, on CD5+ B-1 cell–derived NAbs, which we find may offer a possible avenue to modulate the natural antibody repertoire. Future studies would aim to explore whether these fasting-induced repertoire changes are observed in aged mouse cohorts and enhance functional immune protection. Mechanistic studies investigating the roles of autophagy, metabolic signaling, hormonal regulation, and microenvironmental cues in shaping CD5+ B-1 cell maintenance and repertoire remodeling are critical. Collectively, these results provide a framework for understanding how intermittent fasting can modulate innate-like immunity in a sex- and compartment-specific manner, with potential implications for preserving protective natural antibody responses across the lifespan. Taken together, our data support a model in which intermittent fasting alters the metabolic and antigenic environment experienced by PtC-specific CD5+ B-1 cells, resulting in repertoire alterations. The precise mechanisms responsible for these changes remain to be determined.

## Supplementary Material

vlag038_Supplementary_Data

## Data Availability

The sequencing data were deposited into BankIt (www.ncbi.nlm.nih.gov/WebSub/) with the following submission numbers: 3058113 = Female PerC Fast; 3058114 = Female PerC NF; 3058118 = Female Spleen NF; 3058121 = Female Spleen Fast; 3058124 = Male PerC NF; 3058126 = Male PerC Fast; 3058132 = Male Spleen NF; 3058134 = Male Spleen Fast. All other datasets generated during and/or analyzed during the current study are available on September 9, 2026, in Mendeley Data. The dataset is entitled “Mouse IF Data June 2026” (https://data.mendeley.com/).
