# Supplementary material for: Intermittent fasting alters the antigen-specific CD5+ B-1 cell natural antibody repertoire in male and female mice
Source: Immunohorizons. 2026 Jul 17;10(7):vlag038. doi: 10.1093/immhor/vlag038 (PMC13378454; doi:10.1093/immhor/vlag038)
Supplement: vlag038_Supplementary_Data [file vlag038_supplementary_data.pdf]

A

Male
Peritoneal Washout

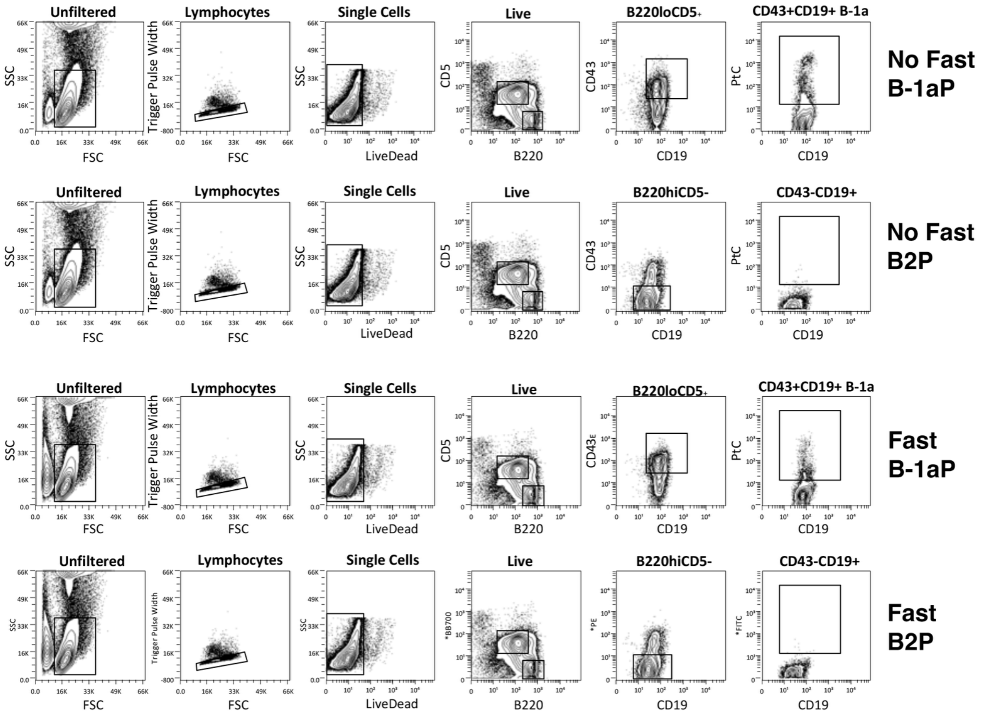

B

Male
Spleen

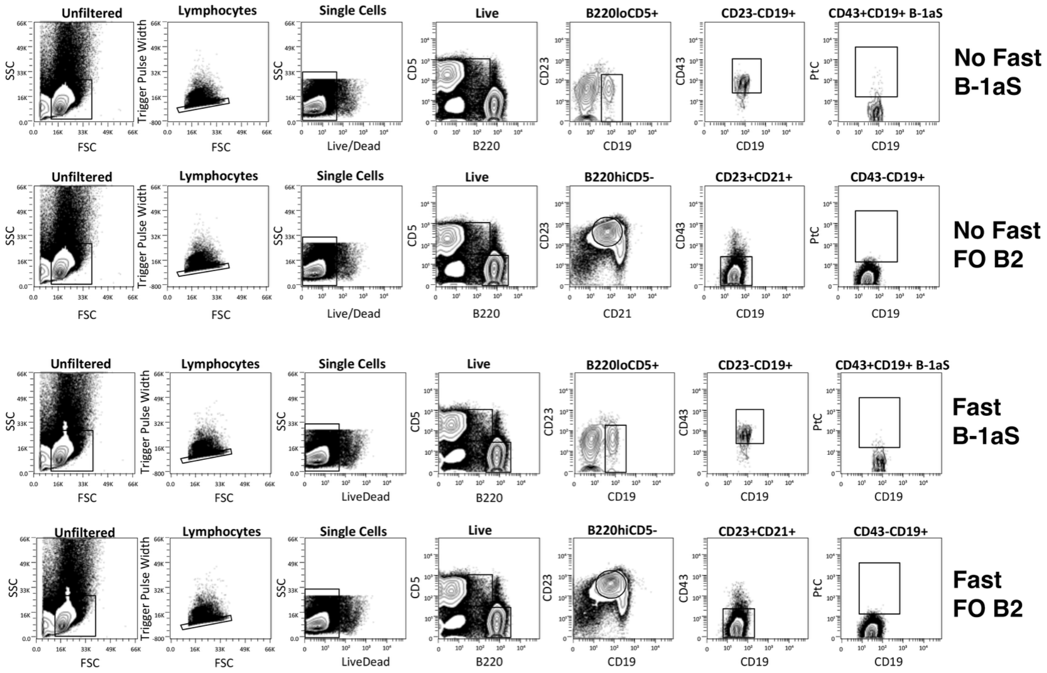

Supplemental Figure 1: Representative gating for peritoneal and splenic PtC+CD5+ B-1 cells. (A) Representative flow plot for gating of peritoneal cavity PtC+CD5+ B-1 cells ($B220^{lo}$, $CD5^{+}$, $CD43^{+}$, $CD19^{+}$, $CD23^{-}$, PtC^{+}) examined in young (4-month-old) non-fasted and fasted mice. The representative plots were obtained from a male mouse peritoneal cavity washout. The internal peritoneal B2 cell population ($B220^{hi}$, $CD5^{-}$, $CD43^{-}$, $CD19^{+}$, $CD23^{+}$) serves as a negative control for PtC gating. **(B)** Representative flow plot for gating of splenic PtC+CD5+ B-1 cells ($B220^{lo}CD5^{+}CD43^{+}CD23^{-}CD19^{+}PtC^{+}$) examined in young (4-month-old) non-fasted and fasted mice. The representative plots were obtained from a male mouse spleen. The internal splenic follicular B2 cell population ($B220^{hi}$, $CD5^{-}$, $CD43^{-}$, $CD19^{+}$, $CD21^{+}$, $CD23^{+}$) serves as a negative control for PtC gating.

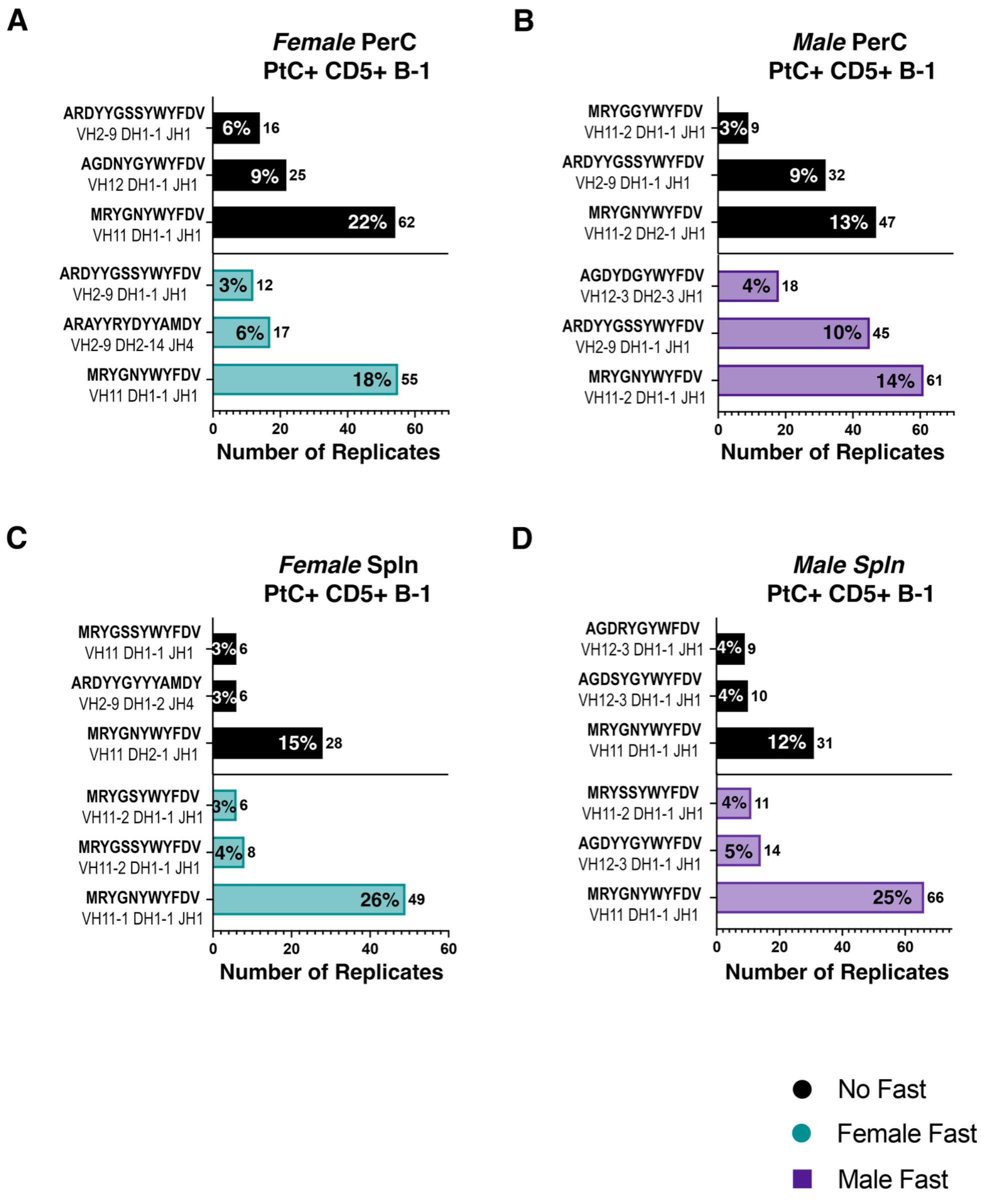
A
Female PerC
PtC+ CD5+ B-1
ARDYYGSSYWYFDV
VH2-9 DH1-1 JH1
6% 16
AGDNYGYWYFDV
VH12 DH1-1 JH1
9% 25
MRYGNYWYFDV
VH11 DH1-1 JH1
22% 62
ARDYYGSSYWYFDV
VH2-9 DH1-1 JH1
3% 12
ARAYYRYDYYAMDY
VH2-9 DH2-14 JH4
6% 17
MRYGNYWYFDV
VH11 DH1-1 JH1
18% 55
0 20 40 60
Number of Replicates
B
Male PerC
PtC+ CD5+ B-1
MRYGGYWYFDV
VH11-2 DH1-1 JH1
3% 9
ARDYYGSSYWYFDV
VH2-9 DH1-1 JH1
9% 32
MRYGNYWYFDV
VH11-2 DH2-1 JH1
13% 47
AGDYDGYWYFDV
VH12-3 DH2-3 JH1
4% 18
ARDYYGSSYWYFDV
VH2-9 DH1-1 JH1
10% 45
MRYGNYWYFDV
VH11-2 DH1-1 JH1
14% 61
0 20 40 60
Number of Replicates
C
Female Spln
PtC+ CD5+ B-1
MRYGSSYWYFDV
VH11 DH1-1 JH1
3% 6
ARDYYGYYYAMDY
VH2-9 DH1-2 JH4
3% 6
MRYGNYWYFDV
VH11 DH2-1 JH1
15% 28
MRYGSYWYFDV
VH11-2 DH1-1 JH1
3% 6
MRYGSSYWYFDV
VH11-2 DH1-1 JH1
4% 8
MRYGNYWYFDV
VH11-1 DH1-1 JH1
26% 49
0 20 40 60
Number of Replicates
D
Male Spln
PtC+ CD5+ B-1
AGDRYGYWFDV
VH12-3 DH1-1 JH1
4% 9
AGDSYGYWYFDV
VH12-3 DH1-1 JH1
4% 10
MRYGNYWYFDV
VH11 DH1-1 JH1
12% 31
MRYSSYWYFDV
VH11-2 DH1-1 JH1
4% 11
AGDYYGYWYFDV
VH12-3 DH1-1 JH1
5% 14
MRYGNYWYFDV
VH11 DH1-1 JH1
25% 66
0 20 40 60
Number of Replicates
No Fast
Female Fast
Male Fast

Supplemental Figure 2: Frequently utilized CDR-H3 Sequences in PtC-specific CD5+ B-1 cells in the absence or presence of intermittent fasting. Young (4-week-old) male (graphs with purple bars) and female (graphs with teal bars) BALB/c-ByJ mice were fasted (as described in Figure 1 and materials and methods) for 12-weeks. Peritoneal cavity washouts and spleens were harvested from the euthanized mice. Single cell suspensions were made, RBCs lysed, and then PtC-specific CD5+ B-1 cells ($B220^{lo}$, $CD5^{+}$, $CD43^{+}$, $CD19^{+}$, $CD23^{-}$, PtC^{+}) were single-cell sorted into a 96-well plate with cell lysis buffer. The V_H region was amplified and sequenced. **(A)** Comparison of the most frequently utilized CDR-H3 sequences of peritoneal PtC+CD5+ B-1 cells from young female mice. **(B)** Comparison of the most frequently utilized CDR-H3 sequences of peritoneal PtC+CD5+ B-1 cells from young male mice. **(D)** Comparison of the most frequently utilized CDR-H3 sequences of splenic PtC+CD5+ B-1 cells from young female mice. **(E)** Comparison of the most frequently utilized CDR-H3 sequences of splenic PtC+CD5+ B-1 cells from young male mice. Results based on three independent experiments for male mice (n=15 mice total per group = non-fast vs. fast, 5 mice/experiment/group) and separately, two independent experiments for female mice (n=10 mice total per group = non-fast vs. fast, 5 mice/experiment/group).

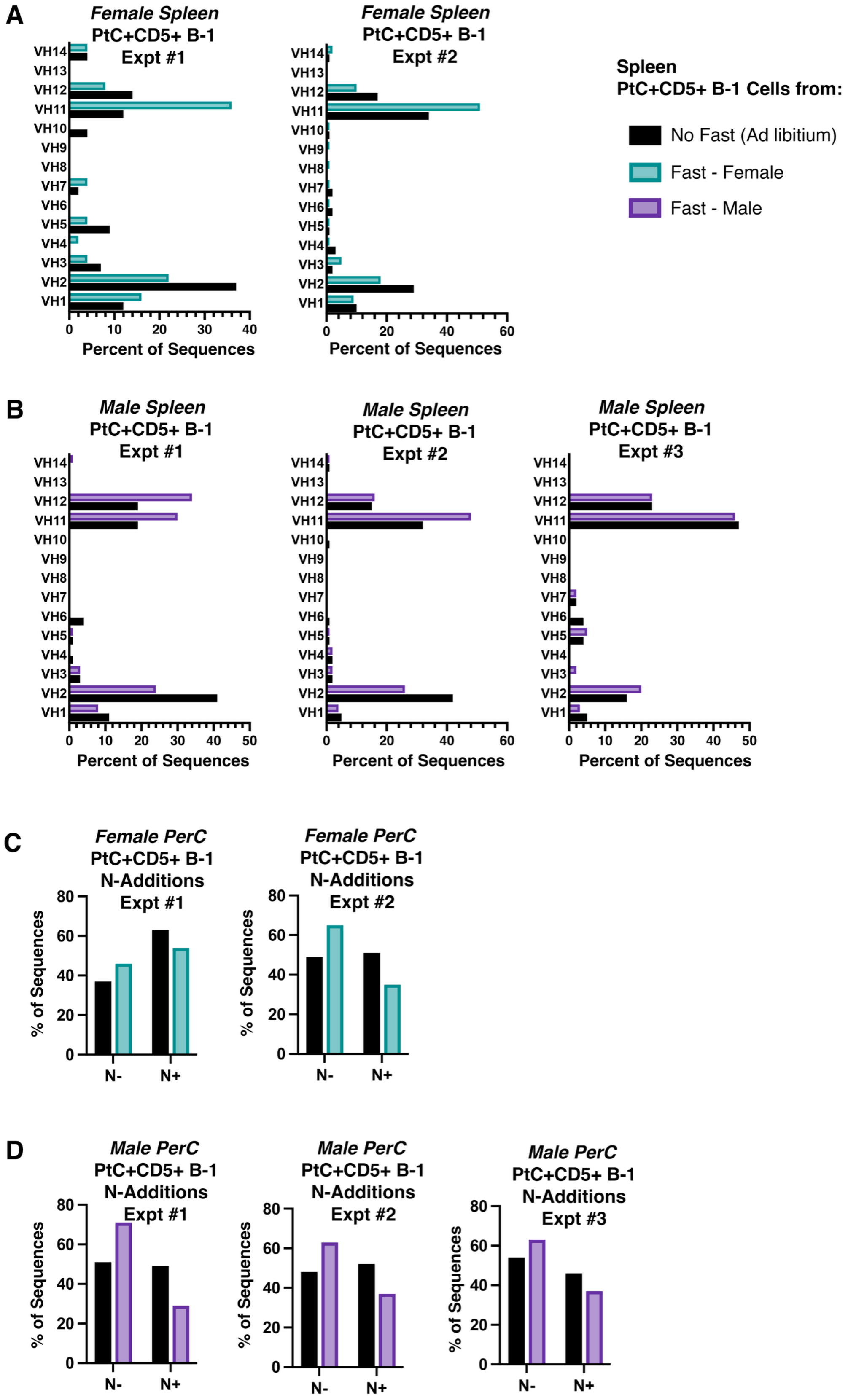

A
Female Spleen
PtC+CD5+ B-1
Expt #1
VH14
VH13
VH12
VH11
VH10
VH9
VH8
VH7
VH6
VH5
VH4
VH3
VH2
VH1
0
10
20
30
40
Percent of Sequences
Female Spleen
PtC+CD5+ B-1
Expt #2
0
20
40
60
Percent of Sequences
Spleen
PtC+CD5+ B-1 Cells from:
No Fast (Ad libitium)
Fast - Female
Fast - Male
B
Male Spleen
PtC+CD5+ B-1
Expt #1
0
10
20
30
40
50
Percent of Sequences
Male Spleen
PtC+CD5+ B-1
Expt #2
0
20
40
60
Percent of Sequences
Male Spleen
PtC+CD5+ B-1
Expt #3
0
10
20
30
40
50
Percent of Sequences
C
Female PerC
PtC+CD5+ B-1
N-Additions
Expt #1
% of Sequences
80
60
40
20
0
N-
N+
Female PerC
PtC+CD5+ B-1
N-Additions
Expt #2
D
Male PerC
PtC+CD5+ B-1
N-Additions
Expt #1
Male PerC
PtC+CD5+ B-1
N-Additions
Expt #2
Male PerC
PtC+CD5+ B-1
N-Additions
Expt #3

Supplemental Figure 3: Repertoire analysis of splenic PtC-specific CD5+ B-1 cells in the absence or presence of intermittent fasting: Individual Experiment Analysis. The individual experiments that make up the VH and N-addition data shown in Figure 3 are presented. Young (4-week-old) male (graphs with purple bars) and female (graphs with teal bars) BALB/c-ByJ mice were fasted (as described in Figure 1 and materials and methods) for 12-weeks. Spleens were harvested from the euthanized mice. Single cell suspensions were made, RBCs lysed, and then PtC-specific CD5+ B-1 cells ($B220^{lo}$, $CD5^{+}$, $CD43^{+}$, $CD19^{+}$, $CD23^{-}$, PtC^{+}) were single-cell sorted into a 96-well plate with cell lysis buffer. The V_H region was amplified and sequenced. **(A)** The percent of V_H gene segment usage for the two independent experiments performed in female mice. **(B)** The percent of V_H gene segment usage for the three independent experiments performed in male mice. **(C)** The number of N-additions at both junctions for the two independent experiments performed in female mice. **(D)** The number of N-additions at both junctions for the three independent experiments performed in male mice. Mice were pooled within each experiment prior to single-cell sorting; therefore, each panel represents one independent pooled cohort. These data are presented as a reproducibility analysis rather than as independently powered statistical comparisons. Three independent experiments for male mice (n=15 mice total per group = non-fast vs. fast, 5 mice/experiment/group) and separately, two independent experiments for female mice (n=10 mice total per group = non-fast vs. fast, 5 mice/experiment/group).
